# Epigenetically associated IGF2BP3 upregulation promotes cell proliferation by regulating E2F1 expression in hepatocellular carcinoma

**DOI:** 10.1038/s41598-024-67021-w

**Published:** 2024-07-11

**Authors:** Chenghao Liu, Yicheng Zhuo, Xiaofeng Yang, Chen Yang, Min Shu, Bowen Hou, Jun Hou, Xueling Chen, Lianghai Wang, Xiangwei Wu

**Affiliations:** 1 NHC Key Laboratory of Prevention and Treatment of Central Asia High Incidence Diseases, Shihezi University, Shihezi, Xinjiang China; 2 Key Laboratory of Xinjiang Endemic and Ethnic Diseases, Shihezi University School of Medicine, Shihezi, Xinjiang China

**Keywords:** E2F1, HCC, Methylation, Prognosis, RNA-binding protein, Hepatocellular carcinoma, Oncogenesis

## Abstract

RNA-binding proteins (RBPs) are a class of proteins that primarily function by interacting with different types of RNAs and play a critical role in regulating the transcription and translation of cancer-related genes. However, their role in the progression of hepatocellular carcinoma (HCC) remains unclear. In this study, we analyzed RNA sequencing data and the corresponding clinical information of patients with HCC to screen for prognostic RBPs. Insulin-like growth factor 2 mRNA-binding protein 3 (IGF2BP3) was identified as an independent prognostic factor for liver cancer. It is upregulated in HCC and is associated with a poor prognosis. Elevated IGF2BP3 expression was validated via immunohistochemical analysis using a tissue microarray of patients with HCC. *IGF2BP3* knockdown inhibited the proliferation of Hep3B and HepG2 cells, whereas *IGF2BP3* overexpression promoted the expansion of HuH-7 and MHCC97H cells. Mechanistically, IGF2BP3 modulates cell proliferation by regulating *E2F1* expression. DNA hypomethylation of the *IGF2BP3* gene may increase the expression of IGF2BP3, thereby enhancing cell proliferation in HCC. Therefore, IGF2BP3 may act as a novel prognostic biomarker and a potential therapeutic target for HCC.

## Introduction

Primary liver cancer, mainly classified as hepatocellular carcinoma (HCC), is a malignancy with high prevalence and a poor prognosis worldwide^1^. Due to high rates of recurrence, low detection rates of curable stages, and ineffective medical treatment, patients with HCC usually do not have a good prognosis^2^. The pathogenesis of HCC is considered a multistep, long-term progressive process^3^; however, the exact molecular mechanism underlying its pathogenesis remains unclear. Therefore, new tumor regulators should be identified, and potential mechanisms underlying tumor growth should be elucidated to identify novel biomarkers and therapeutic targets to treat HCC early.

The primary regulators of the mRNA life cycle are RNA-binding proteins (RBPs) and microRNAs^4^. RBPs bind to their targets and orchestrate various aspects of RNA dynamics, including subcellular localization, translational efficiency, and RNA metabolism^5,6^. Over 1500 genes encoding RBPs have been identified in the human genome through genome-wide screens^7^. Physiological processes depend on these RBPs, especially during cell development and stress responses^8^. Therefore, abnormal changes in the expression or functions of these proteins may contribute to the development of various diseases, such as cancer^6^. Although studies have indicated that RBPs play a pivotal role in the development and progression of multiple malignancies^9^, their role in HCC remains unclear.

Insulin-like growth factor 2 mRNA-binding proteins (IGF2BPs) are a family of highly conserved mRNA-binding proteins and novel m6A readers that protect mRNAs modified by m6A from degradation^10^. The IGF2BP family comprises three RBPs, namely, IGF2BP1, IGF2BP2, and IGF2BP3^11^. IGF2BP1 has been extensively studied in various cellular experiments; however, relatively few functional studies have examined the role of IGF2BP3^11^. IGF2BP3 is an oncoprotein abnormally expressed in several malignancies, such as lung, gastrointestinal, and ovarian cancers^12–14^. It is supposed to be associated with tumor cell proliferation, survival, invasion, and resistance to therapeutic drugs^14–16^.

In this study, the expression profiles of all known RBPs were examined to identify RBPs associated with the progression of liver cancer. IGF2BP3 expression was associated with a shorter survival time in HCC. *IGF2BP3* knockdown inhibited, while *IGF2BP3* overexpression promoted the proliferation of tumor cells. IGF2BP3 was found to enhance cell proliferation by regulating the expression of E2F1.

## Results

### Selection of prognosis-related RBPs in HCC

**Figure 1:**
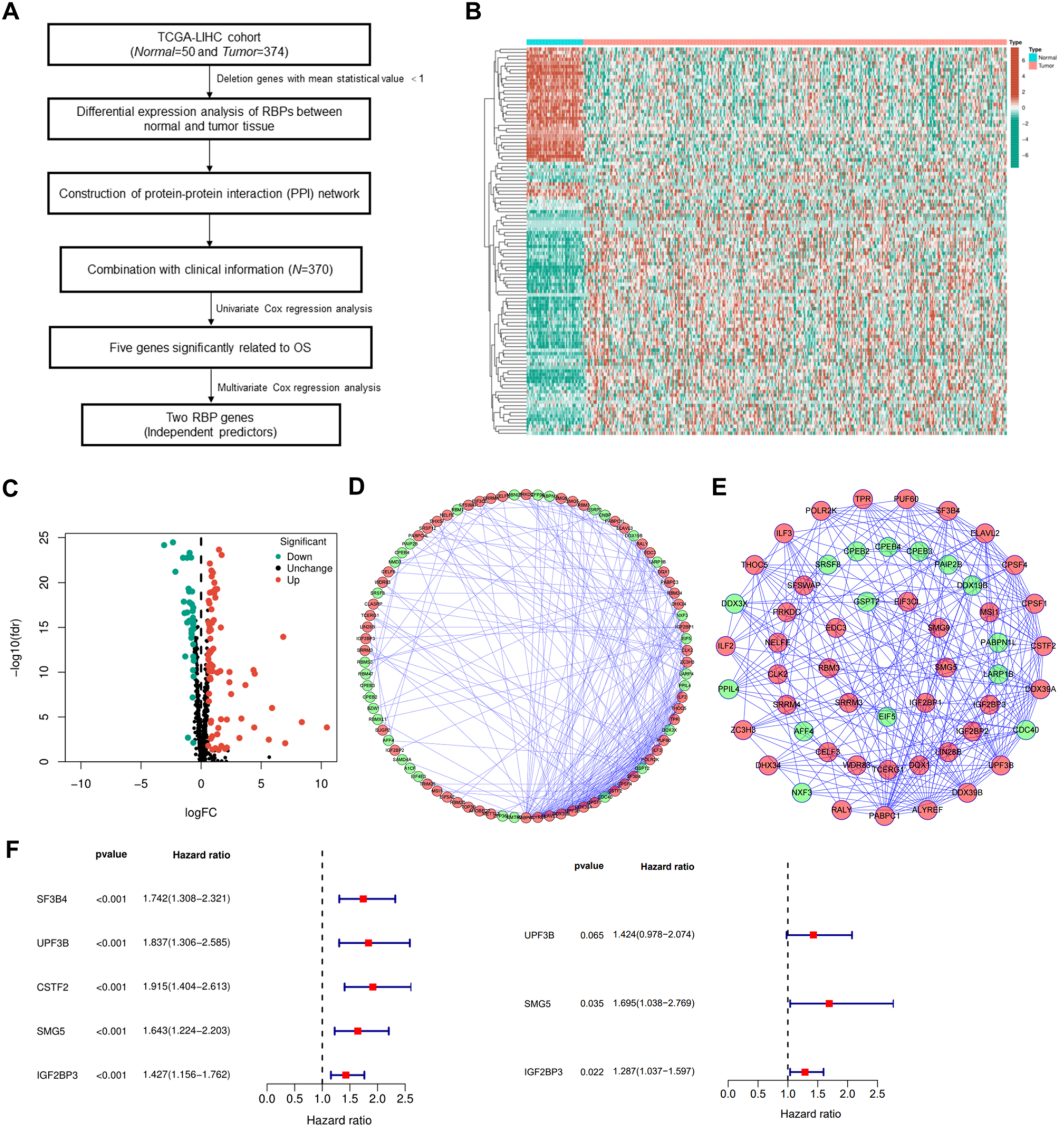
Selection of prognosis-related RBPs in HCC. (**A**) Flowchart demonstrating the workflow of identification of critical prognostic RBPs. (**B**) Heatmap of differentially expressed RBPs. (**C**) Volcano plot of differentially expressed RBPs. Upregulated and downregulated genes are represented in red and green, respectively. FDR, false discovery rate. (**D**,**E**) PPI network of all and core module-related differentially expressed RBPs (Interaction Score > 0.4) and subnetworks built through the MCODE plugin (Degree Cutoff = 2). The density of the lines represents the ability to interact. Red and green nodes represent upregulated and downregulated RBPs, respectively. (**F**) Univariate and multivariate Cox regression analyses for identification of critical prognosis-related RBPs.

A flowchart demonstrating the study design is shown in Fig. 1A. The gene expression data of 374 tumors and 50 normal liver samples were extracted from TCGA-LIHC cohort. A total of 692 RBP-associated genes^7^ were selected for the subsequent screening of differentially expressed RBPs. Based on the threshold of FDRs < 0.05 and |log2 fold change| values > 0.585, 112 (74 upregulated and 38 downregulated) RBPs were identified between HCC and adjacent non-tumor tissues (Fig. 1B and C). To examine the potential function of the identified RBPs, they were ranked based on fold change values and annotated using the clusterProfiler R package. GO enrichment analysis revealed that the differentially expressed RBPs were significantly involved in pathways related to the regulation of mRNA metabolic processes, cytoplasmic ribonucleoprotein granules, and translation regulator activity (Supplementary Table 3). KEGG enrichment analysis revealed that the differentially expressed RBPs mainly promoted the mRNA surveillance pathway and RNA transport (Supplementary Table 4). Additionally, a PPI network of these RBPs was constructed using the MCODE tool in Cytoscape, and the top module with the best interaction score was found (Fig. 1D and E). A total of 53 core module-related RBPs were identified from the PPI network. Univariate (*P* < 0.001) and multivariate (*P* < 0.05) Cox regression analyses were performed to investigate the prognostic significance of these RBPs, and two prognostic RBPs were identified, namely, SMG5 and IGF2BP3 (Fig. 1F).

### IGF2BP3 is a potential marker for the prognosis of HCC

**Figure 2:**
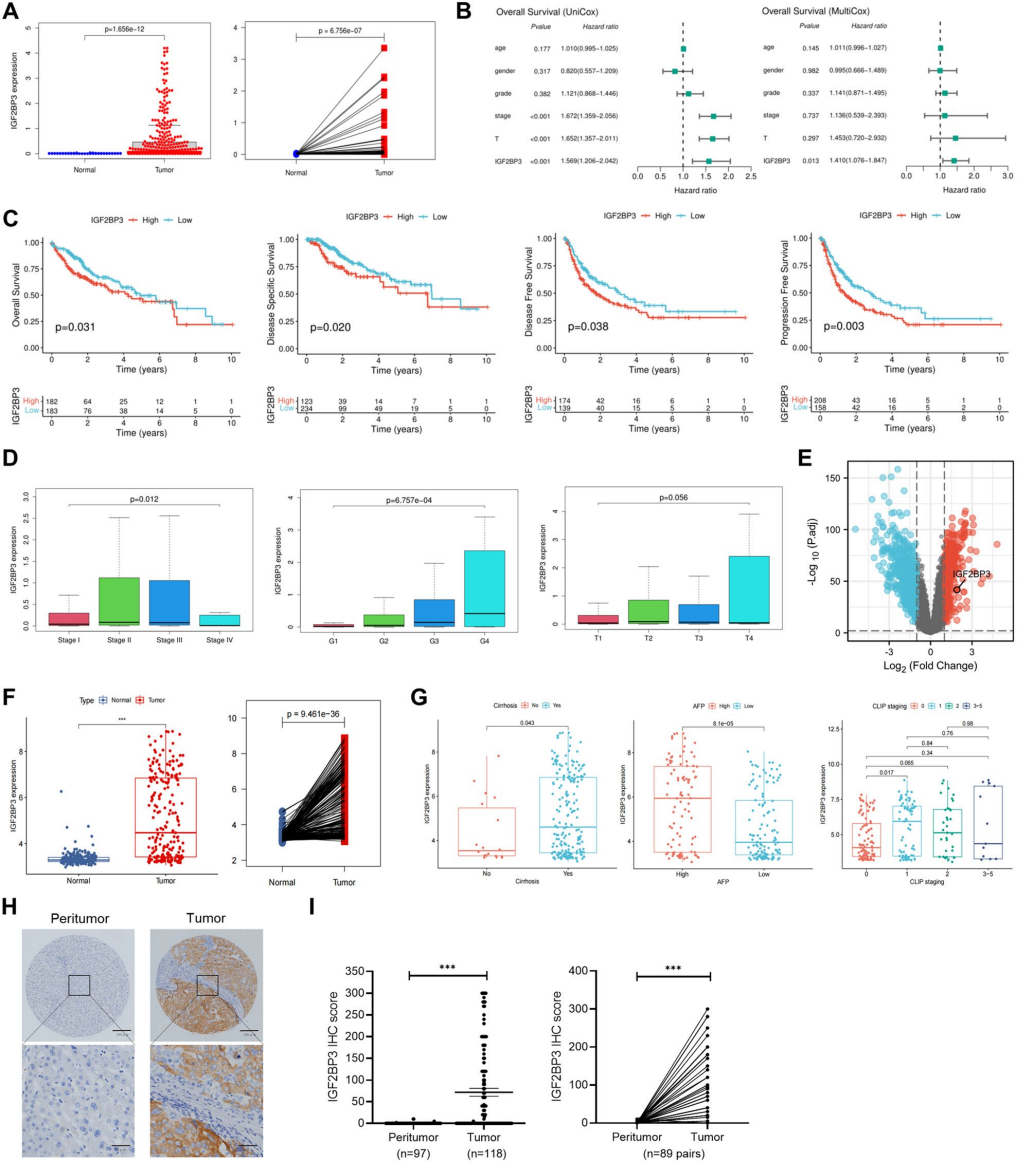
IGF2BP3 is a potential marker for the prognosis of HCC. (**A**) *IGF2BP3* expression in unpaired (left) and paired (right) HCC and normal tissues in TCGA-LIHC dataset. (**B**) Univariate (left) and multivariate (right) Cox regression analyses for *IGF2BP3*. (**C**) Kaplan–Meier analysis for examining the correlation between *IGF2BP3* expression and overall, disease-specific, disease-free, and progression-free survival in TCGA-LIHC dataset. (**D**) Correlation between *IGF2BP3* expression and clinical characteristics of patients in TCGA-LIHC dataset. (**E**) Volcano plot of differentially expressed RBPs in the GSE14520 dataset. Upregulated and downregulated genes are represented in red and blue, respectively. (**F**) *IGF2BP3* expression in unpaired (left) and paired (right) HCC and normal tissues in the GSE14520 dataset. (**G**) Correlation between *IGF2BP3* expression and clinical characteristics of patients with HCC in the GSE14520 dataset. (**H**) Representative images of immunohistochemical staining of IGF2BP3 in peri-tumor (left) and tumor (right) tissues. (**I**) Statistical analysis of immunohistochemical scores in unpaired (left) or paired (right) tumor and peri-tumor tissues. Significant differences were estimated using the Mann–Whitney test ((**I**), left) and the Wilcoxon matched-pairs signed-rank test ((**I**), right).

Of the two identified prognostic RBPs, IGF2BP3 was selected for subsequent analysis because its role as a member of the m6A reader family in HCC remains unclear. *IGF2BP3* expression was significantly higher in tumors than in normal liver tissues in TCGA-LIHC cohort (Fig. 2A). After adjusting for clinical variables, including age, sex, tumor grade, pathological stage, and T stage, univariate (*P* < 0.001) and multivariate (*P* < 0.05) Cox analyses showed that *IGF2BP3* was an independent predictor of overall survival (Fig. 2B). Furthermore, Kaplan–Meier analysis assessed the relationship between *IGF2BP3* expression and survival in HCC. Patients with high *IGF2BP3* expression had shorter overall, disease-specific, disease-free, and progression-free survival (Fig. 2C). Additionally, we examined the association between *IGF2BP3* expression and the clinical characteristics of patients with HCC. High *IGF2BP3* expression was significantly correlated with the advanced pathological stages and tumor grades (Fig. 2D). The dysregulated expression of *IGF2BP3* was further validated in the independent dataset GSE14520 (Fig. 2E,F). Patients with HCC with cirrhosis, high AFP levels, and advanced CLIP stages had higher *IGF2BP3* expression (Fig. 2G). Furthermore, immunohistochemical (IHC) staining was performed to determine the expression of IGF2BP3 in HCC and peri-tumor tissues. As shown in Fig. 2H, IGF2BP3 was primarily found in the cytoplasm of HCC tissues, and its expression was significantly higher in cancer specimens than in peri-tumor tissues. Of 118 samples, 59 (50%) showed positive expression of IGF2BP3, whereas the respective peri-tumor tissues did not express IGF2BP3 (*P* < 0.001; Fig 2I). Patients in the cohort were divided into low- and high-*IGF2BP3*-expression groups based on the median expression level of *IGF2BP3*. High *IGF2BP3* expression was associated with liver capsule invasion and increased AFP levels (Table 1). Altogether, upregulation of IGF2BP3 was associated with a poor prognosis of HCC.

**Table 1:** Correlation between IGF2BP3 expression and clinical characteristics of patients with HCC.

Clinical characteristic	IGF2BP3^High^ (n)	IGF2BP3^Low^ (n)	*P-*value
Age (years)			0.847
< 60	34	38	
≥ 60	14	17	
Sex			0.578
Men	43	43	
Women	10	13	
Tumor size (cm)			0.251
< 5	23	32	
≥ 5	23	20	
Tumor number			0.468
Single	42	47	
Multiple	7	5	
TNM stage			0.028*
I–II	34	44	
III–IV	13	5	
Microvascular invasion			0.315
No	27	35	
Yes	22	19	
Liver capsule invasion			0.022*
No	26	40	
Yes	16	8	
Lymphoid infiltrates			0.067
No	26	33	
Yes	5	1	
Cirrhosis			0.451
No	7	10	
Yes	43	41	
HBsAg			0.565
No	6	8	
Yes	45	43	
AFP levels			0.032*
< 200 ng/mL	22	31	
≥ 200 ng/mL	29	17	
Distant metastases			0.394
No	44	50	
Yes	5	3	
Lymphatic metastasis			0.583
No	46	51	
Yes	3	2	

Significant values are in bold. *P < 0.05.

### IGF2BP3 promotes the proliferation of HCC cells

**Figure 3:**
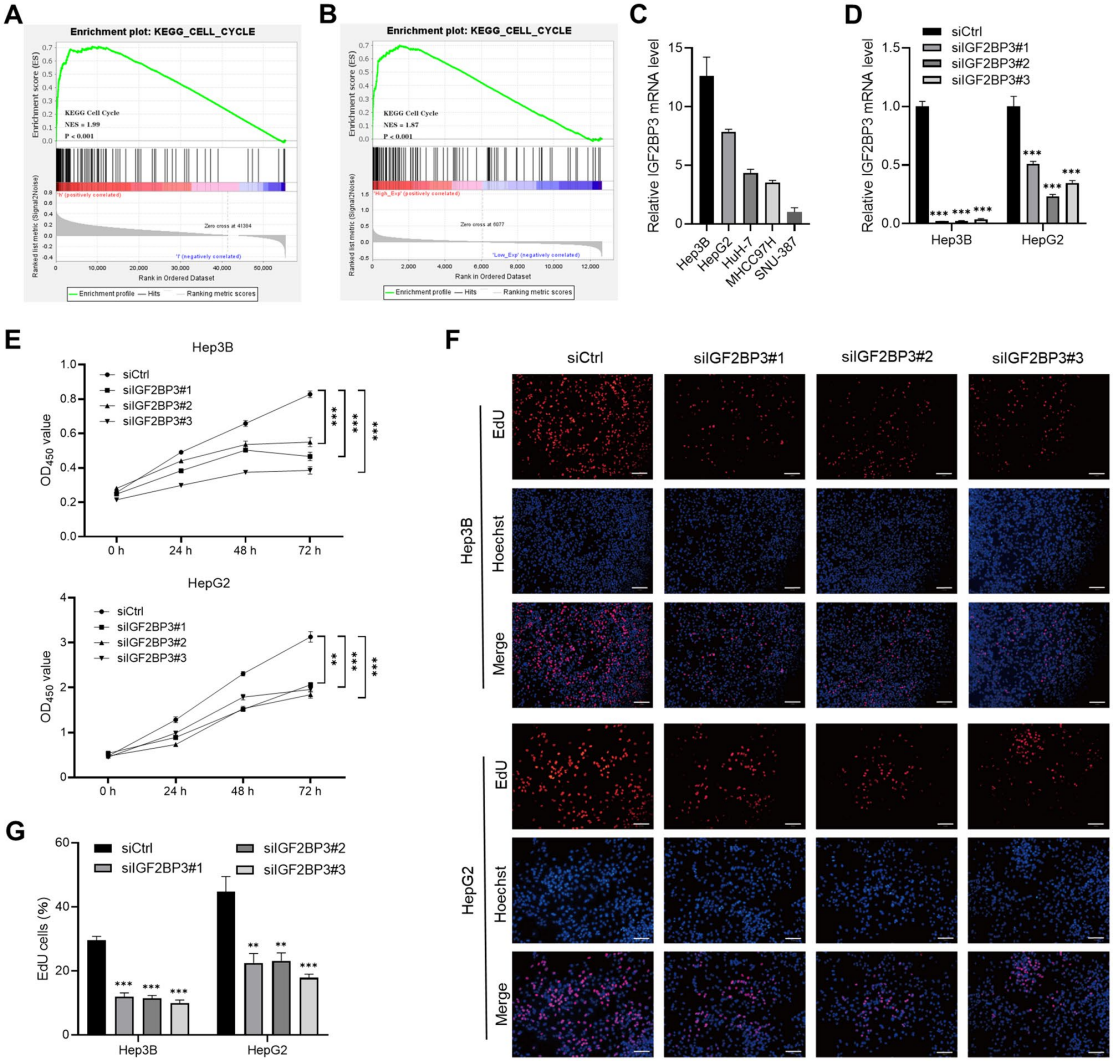
Silencing of IGF2BP3 inhibits the proliferation of HCC cells. (**A**,**B**) Gene set enrichment analysis was used to evaluate the enrichment scores of the indicated gene set^37^ in the high- and low-*IGF2BP3*-expression groups in TCGA-LIHC and GSE14520 cohorts. NES, normalized enrichment score; FDR, false discovery rate. (**C**) qRT-PCR was used to assess *IGF2BP3* expression in hepatic tumor cells. (**D**) qRT-PCR validated the siRNA-mediated knockdown of *IGF2BP3* in Hep3B and HepG2 cells. (**E**) CCK-8 assay revealed that siRNA-mediated downregulation of *IGF2BP3* significantly decreased the growth rate of Hep3B (top) and HepG2 (bottom) cells. (F, G) Representative micrographs and quantification of EdU incorporation in the indicated cells. Significant differences were estimated via two-way ANOVA (**E**) or one-way ANOVA with Turkey post hoc tests (**D**,**G**).

GSEA was used to explore the potential role of IGF2BP3 in the progression of HCC. Cell cycle-related genes were significantly enriched in the high-*IGF2BP3*-expression group in both TCGA-LIHC (NES = 1.99, *P* < 0.001; Fig. 3A) and GSE14520 (NES = 1.87, *P* < 0.001; Fig. 3B) datasets. qRT-PCR was performed to analyze *IGF2BP3* expression in several human hepatic tumor cell lines, and the results revealed that *IGF2BP3* expression was high in Hep3B and HepG2 cells (Fig. 3C). Furthermore, functional experiments were performed after knocking down *IGF2BP3* via siRNAs in Hep3B and HepG2 cells (Fig. 3D). CCK8 assay showed that siRNA-mediated downregulation of *IGF2BP3* significantly decreased the growth rate of Hep3B and HepG2 cells (Fig. 3E) and the proportion of EdU-positive cells (Fig. 3F,G).

**Figure 4:**
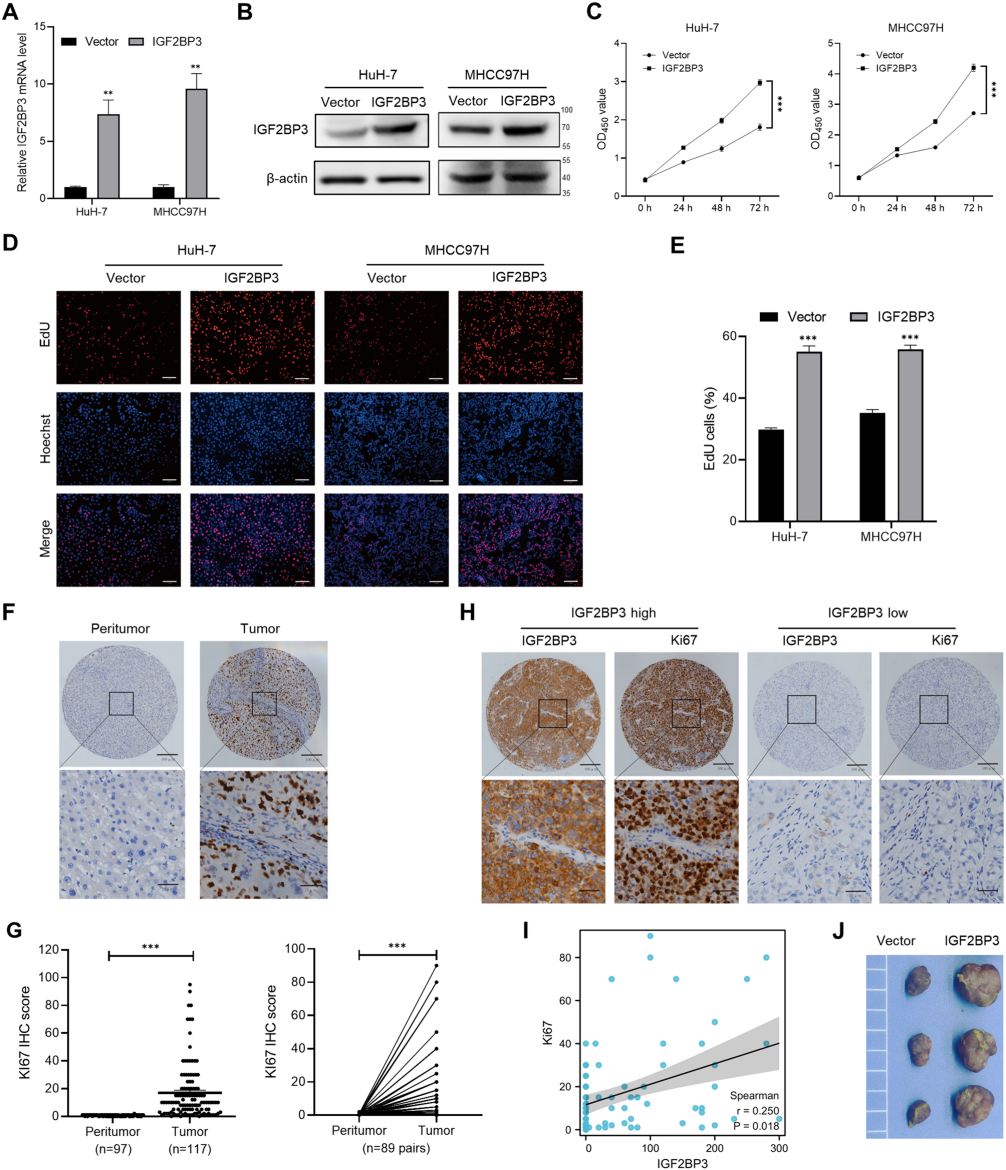
IGF2BP3 promotes the proliferation of HCC cells. (**A**,**B**) qRT-PCR and western blotting were performed to verify the upregulation of IGF2BP3 in HuH-7 and MHCC97H cells after ectopic expression of *IGF2BP3*. (**C**) CCK-8 assay revealed that upregulation of *IGF2BP3* via transduction with a recombinant lentivirus significantly increased the growth rate of HuH-7 (left) and MHCC97H (right) cells. (**D**,**E**) Representative micrographs and quantification of EdU incorporation in the indicated cells. (**F**) Representative images of immunohistochemical staining of Ki67 in peri-tumor (left) and tumor (right) tissues. (**G**) Statistical analysis of immunohistochemical scores in unpaired (left) or paired (right) tumor and peri-tumor tissues. (**H**) Representative immunohistochemical staining of IGF2BP3 (left) and Ki67 (right) expression in HCC samples. (**I**) A scatter plot showing the correlation between the IHC scores of IGF2BP3 and Ki67. (**J**) Tumors formed by subcutaneous injection of HuH-7 cells with IGF2BP3 overexpression or vector control in nude mice. Significant differences were estimated using unpaired Student’s *t*-test (**A**,**E**), two-way ANOVA (**C**), Mann–Whitney test ((**G**), left), and Wilcoxon matched-pairs signed-rank test ((**G**), right).

Recombinant lentiviruses successfully induced *IGF2BP3* expression in HuH-7 and MHCC97H cells (Fig. 4A,B). Overexpression of *IGF2BP3* significantly increased the growth rate of HuH-7 and MHCC97H cells (Fig. 4C) and the proportion of EdU-positive cells (Fig. 4D,E). Additionally, the expression of Ki67, a proliferation indicator, was analyzed in HCC tissue samples to examine the correlation between IGF2BP3 and cell proliferation (Fig. 4F,G). The proliferation capacity was correlated with high IGF2BP3 expression in tumor tissues (r = 0.25, *P* < 0.05; Fig. 4H,I). When subcutaneously transplanted into nude mice, the growth of HuH-7 cells with *IGF2BP3* overexpression was markedly enhanced compared to control cells (Fig. 4J). These results indicate that IGF2BP3 may be involved in the proliferation of HCC cells.

### IGF2BP3 modulates cell proliferation by regulating E2F1 expression

**Figure 5:**
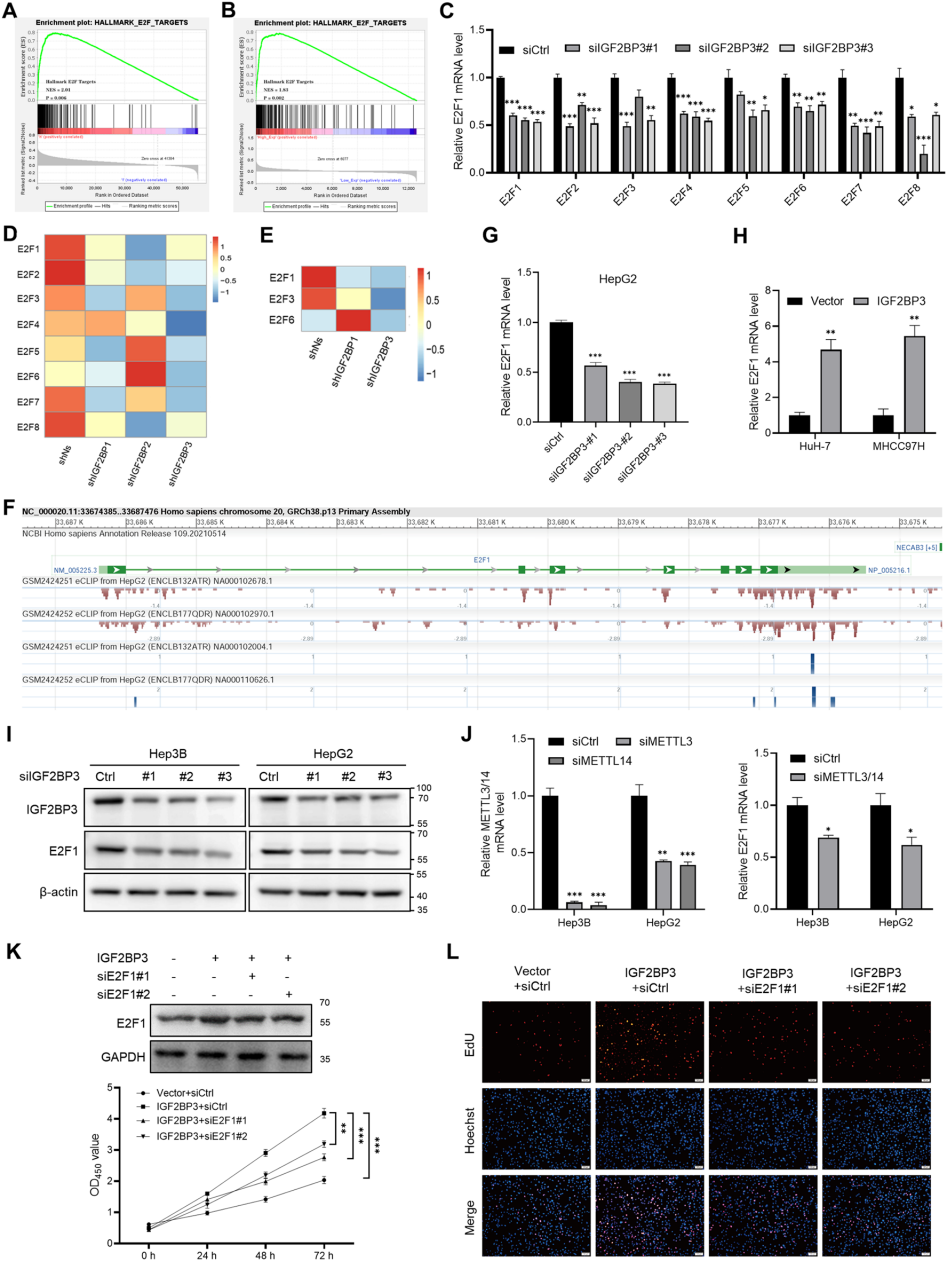
IGF2BP3 modulates cell proliferation by regulating E2F1 expression. (**A**,**B**) Gene set enrichment analysis was used to evaluate the enrichment scores of the indicated gene set in the high- and low-*IGF2BP3*-expression groups in TCGA-LIHC and GSE14520 cohorts. (**C**) qRT-PCR was performed to verify the reduced expression of *E2F1*–*8* in Hep3B cells after *IGF2BP3* knockdown. (**D**,**E**) Heatmap showing reduced gene expression and mRNA half-lives of E2Fs in HepG2 cells with IGF2BP knockdown. (**F**) The enrichment of IGF2BP3 binding peaks in the transcript of *E2F1* derived from GSE92220 (crosslinking and immunoprecipitation of IGF2BP3). (**G**,**H**) qRT-PCR was used to assess *E2F1* expression in the indicated cells. (**I**) Western blotting was used to verify reduced E2F1 expression in Hep3B and HepG2 cells after *IGF2BP3* knockdown. (**J**) qRT-PCR was used to verify reduced *E2F1* expression in Hep3B and HepG2 cells after METTL3/14 knockdown. (**K**,**L**) CCK-8 and EdU assays showed that upregulation of IGF2BP3 significantly enhanced the growth of HuH-7 cells, which could be reversed by E2F1 silencing. Significant differences were estimated using the one-way ANOVA with Turkey post hoc tests (**C**, **G**, and **J**), unpaired Student’s *t*-test (**H** and **J**), and two-way ANOVA (**K**).

The enrichment scores of hallmark gene sets in tumors with varying levels of *IGF2BP3* expression were evaluated to understand the role of IGF2BP3 in regulating cell function in HCC. GSEA showed that E2F targets were significantly enriched in the high-*IGF2BP3*-expression group in both TCGA-LIHC (NES = 2.01, *P* < 0.01) and GSE14520 (NES = 1.83, *P* < 0.01) datasets (Fig. 5A,B). The E2F family comprises eight transcription factors. Since the E2F member involved in IGF2BP3-mediated cell proliferation remains unknown, the prognostic impact of all E2Fs (E2F1–8) was further assessed. Survival data from TCGA-LIHC were used to determine the relationship between the expression of E2Fs and survival. Kaplan–Meier analysis revealed that patients with high expression of *E2F1*–*8* had relatively poor survival (Supplementary Fig. 1). Subsequently, qRT-PCR was performed to examine the mRNA expression of *E2F1*–*8* in Hep3B cells with *IGF2BP3* knockdown. Results showed that the mRNA expression of *E2F1*–*8* was reduced to different degrees after *IGF2BP3* knockdown (Fig. 5C). In addition, a positive correlation was observed between all E2Fs and *IGF2BP3* (Supplementary Fig. 2). To examine the effects of IGF2BPs on mRNA stability and gene expression output, we reanalyzed a publicly available RNA-seq dataset GSE90684 of IGF2BP-knockdown cells and control HepG2 cells with or without actinomycin D treatment^17^. The mRNA expression of *E2F1*–*8* decreased to different degrees after silencing each IGF2BP (Fig. 5D). In addition, the half-lives of *E2F1*, *E2F3,* and *E2F6* significantly decreased in *IGF2BP3*-knockdown cells (Fig. 5E), suggesting that IGF2BP3 is involved in the stabilization of the three E2Fs. Furthermore, the eCLIP experiment on HepG2 cells (GSE92220) identified abundant binding sites for IGF2BP3 on the *E2F1* transcript (Fig. 5F). siRNA-mediated knockdown of *IGF2BP3* significantly reduced E2F1 expression in Hep3B and HepG2 cells (Fig. 5G,I), whereas IGF2BP3 overexpression significantly increased *E2F1* expression in HuH-7 and MHCC97H cells (Fig. 5H). Notably, the knockdown of the m6A writers *METTL3* and *METTL14* also decreased *E2F1* expression in Hep3B and HepG2 cells (Fig. 5J). Furthermore, the silencing of E2F1 significantly reversed the promoting effect of IGF2BP3 on the proliferative capacity in HuH-7 cells (Fig. 5K,L). Therefore, IGF2BP3, as an m6A reader, may regulate E2F1 expression by recognizing m6A on the *E2F1* transcript. E2F transcription factors are the primary regulators of the cell cycle. In particular, E2F1 plays an essential role in G1–S phase transition^18^. Therefore, we hypothesized that IGF2BP3 could influence the cell cycle by regulating E2F1 expression, thus controlling the proliferation of HCC cells.

### DNA methylation is negatively correlated with IGF2BP3 expression

**Figure 6:**
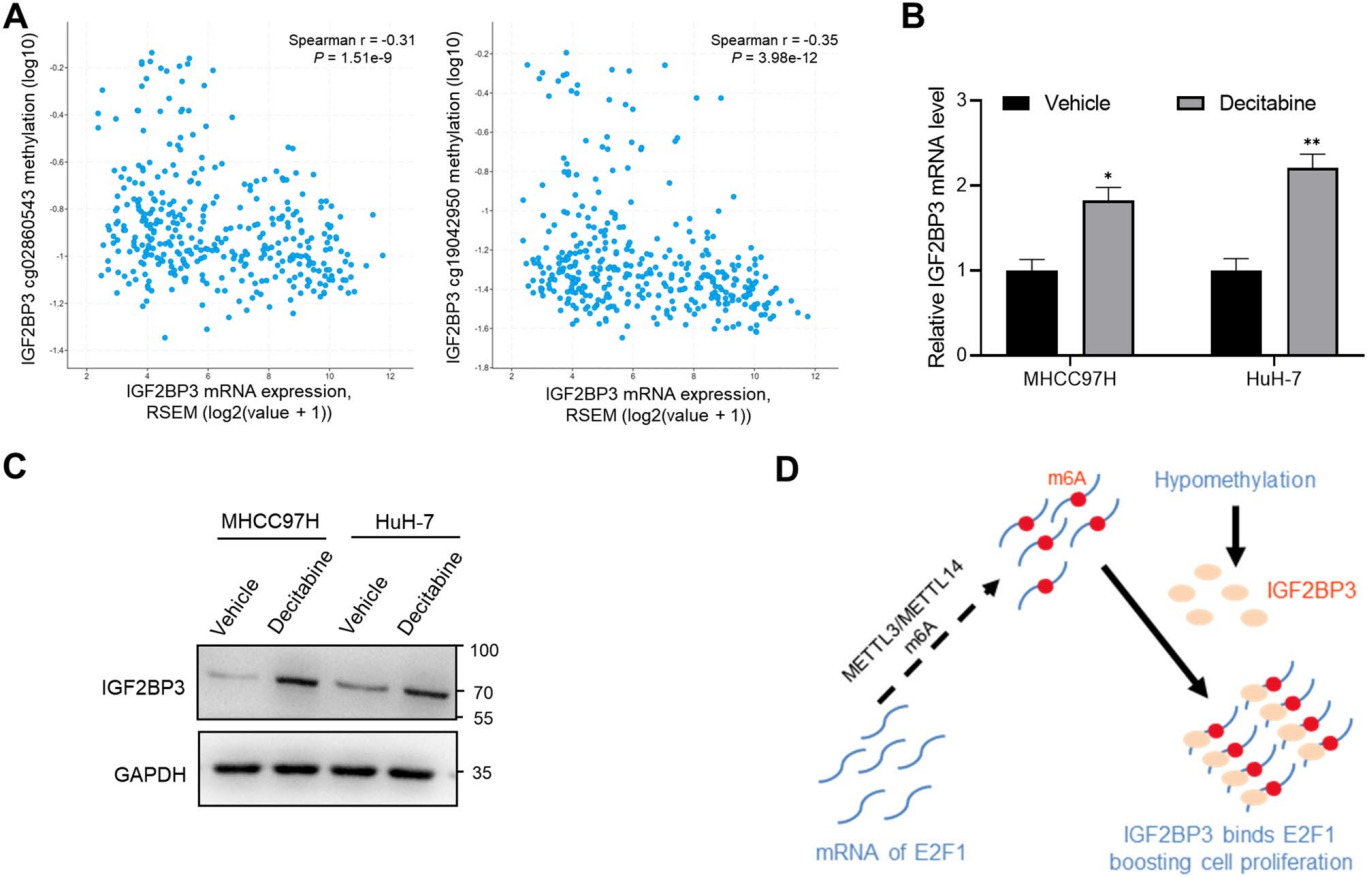
DNA methylation is negatively correlated with IGF2BP3 expression. (**A**) The correlation between *IGF2BP3* expression and methylation status in the promoter regions was visualized using the cBioPortal for Cancer Genomics. Spearman correlation coefficients and *P*-values for *IGF2BP* expression and methylation status are shown. (**B**) qRT-PCR was used to analyze *IGF2BP3* expression in MHCC97H and HuH-7 cells treated with decitabine. Significant differences were estimated using the unpaired Student’s *t*-test. (**C**) Analysis of IGF2BP3 expression in MHCC97H and HuH-7 cells treated with decitabine by western blotting. (**D**) A proposed model for IGF2BP3 promoting tumor proliferation by regulating E2F1.

To understand the mechanisms underlying the aberrant upregulation of IGF2BP3 in HCC tissues, we examined the correlation between the expression and methylation of *IGF2BP3*. A negative correlation was observed between *IGF2BP3* expression and the methylation of multiple sites in the *IGF2BP3* promoter (Fig. 6A). Consistently, treatment with decitabine, a DNA methyltransferase inhibitor^19^, significantly enhanced IGF2BP3 expression at mRNA and protein levels in MHCC97H and HuH-7 cells (Fig. 6B,C).

## Discussion

This study identified 112 critical RBPs involved in the occurrence and progression of HCC through rigorous screening. Functional pathway enrichment analysis demonstrated that the differentially expressed RBPs were significantly enriched in translational regulation, mRNA metabolism, negative translational regulation, and biological processes related to mRNA processing. A PPI network based on these RBPs was constructed, and a module comprising 53 core RBPs was obtained. Among them, several key RBPs have been implicated in tumor development and progression. For example, CSTF2 induces shortening of the 3′-untranslated region (UTR) of *RAC1* in human bladder uroepithelial carcinoma by mediating slow transcription of *RAC1*, thus regulating cell proliferation, migration, and invasion^20^. By regulating alternative splicing, CPSF1 induces cell proliferation, invasion, and apoptosis in the head and neck squamous cell carcinoma^21^.

Core module-related RBPs in the PPI network were subjected to univariate and multivariate Cox regression analyses to assess their biological functions and clinical significance. IGF2BP3 and SMG5 were identified as prognostically relevant core RBPs. IGF2BP3, previously called IMP3^22^, was initially identified as the Vg1-RBP/Vera direct homolog of KOC. It is located on human chromosome 7p15.3 and encodes a 69-kDa protein initially identified based on its abundance in pancreatic cancer^23^. Previous studies have reported that IGF2BP3 plays a vital role in cancers^11,23^. Additionally, IGF2BP3, a modulator of the m6A enzyme, is upregulated and regulates the expression of programmed death ligand-1 (PD-L1) to increase the malignancy of tumor cells^24,25^. A dose-dependent decrease in IGF2BP3 expression induced the arrest of G1–S-phase in leukemic cells. IGF2BP3 enhances malignant behavior by increasing proliferation rates and is correlated with the abundance of leukocytes^11,26^. The MYC-activated *IGF2BP3* gene regulates the proliferation, invasion, and metastasis ability of nasopharyngeal and breast carcinoma cells^27,28^.

In this study, the mechanism of action of IGF2BP3 in HCC was examined via bioinformatic analysis and in vitro experiments. GSEA revealed that genes associated with cell cycle and E2F targets were significantly enriched in the high-*IGF2BP3*-expression group. The E2F family is usually divided into two groups based on their specific and distinct functions: transcriptional activators (E2F1, E2F2, and E2F3a) and transcriptional repressors (E2F3b and E2F4–8). These E2Fs coordinate with each other and synergistically control the oscillatory nature of the cell cycle^29^. By directly binding to and altering the subcellular distribution of cytoplasmic E2F7, suppressor anaphase-promoting complex domain 2 (SAPCD2) promotes neuroblastoma progression by modulating E2F activity and affecting genes involved in the cell cycle and chromosomal instability^30^. E2F1 can promote the proliferation and migration of ovarian cancer cells by regulating stathmin 1 (STMN1) overexpression, and high STMN1 expression in ovarian cancer often indicates a poor prognosis^31^. DTX3 governs the growth of colorectal cancer cells by regulating E2F1 and the downstream genes CDC2 and cyclin D3^32^. Since the transcription factor E2F1, a target of IGF2BP3, can regulate the cell cycle, it may function as a critical mediator of the influence of IGF2BP3 on HCC progression.

METTL3 and 14 act as m6A readers and can mediate m6A modification in *E2F1*^33^. Studies employing high-throughput sequencing have revealed that m6A is particularly enriched in the 3'-UTR and near the stop codon of mRNAs with RRACH-consistent sequences (R corresponds to G or a; H corresponds to a, C or U)^17,34^. However, the mechanism of action of IGF2BPs, a new family of m6A readers, in recognizing and regulating their targets remains to be determined. Recently, Huang et al. reported that IGF2BPs preferentially recognize m6A-modified mRNAs and promote the stability (and possibly translation) of numerous potential mRNA targets in an m6A-dependent manner, thereby globally affecting gene expression^17^. This study revealed that IGF2BP3 may regulate HCC progression by controlling the stability and transcription of *E2F1*.

The regulatory machinery includes epigenetic modifications, post-transcriptional modifications of RNA, and post-translational modifications of the proteome^35^. Disrupting any step in this mechanism can result in abnormal cellular behavior^36^. Epigenetic regulation is an important mechanism underlying *IGF2BP3* expression and warrants further investigation.

In summary, the RNA-binding protein IGF2BP3 plays an essential role in the progression of HCC and may facilitate the proliferation of HCC cells by regulating *E2F1* expression (Fig. 6D). Detailed mechanisms underlying IGF2BP3-mediated regulation of E2F1 through the cell cycle warrant further investigation. The IGF2BP3–E2F1 regulatory axis may serve as a new therapeutic target for HCC.

## Materials and methods

### Ethics declarations

The study was approved by the Ethics Committee of the First Affiliated Hospital of Shihezi University. All procedures were carried out following relevant guidelines and regulations and are reported in accordance with ARRIVE guidelines.

### Data processing

RNA sequencing data of 50 normal liver tissues and 374 tumor specimens and the corresponding clinical data of patients with liver hepatocellular carcinoma (LIHC) were extracted from The Cancer Genome Atlas (TCGA) database (https://portal.gdc.cancer.gov/). The limma package was used to identify differently expressed RBPs based on the threshold of |log2 fold change (FC)|> 0.585 and false discovery rates (FDRs) < 0.05. Genes with an average count value < 1 were excluded.

### Functional enrichment analysis

GO and KEGG^37^ pathway enrichment analyses were performed to comprehensively characterize the biological functions of the differently expressed RBPs. Enrichment analysis was performed using the WEB-based Gene Set Analysis Toolkit (WebGestalt, http://www.webgestalt.org/)^38^, and *P*-values and FDRs of < 0.05 were considered statistically significant. Significant RBPs were submitted to the STRING database (http://www.string-db.org/)^39^ to assess protein–protein interaction (PPI), and the Cytoscape software (version 3.8.2) was used to construct and visualize a PPI network. Key modules and genes in the PPI network were selected using the Molecular Complex Detection (MCODE) plug-in based on the threshold of MCODE score and node count number of > 5^40^.

### Screening of critical genes encoding RBPs

Univariate and multivariate Cox regression analyses were performed to identify prognostic RBPs. Briefly, RBPs in the core modules of the dataset were tested using the survival R package. Variables with a *P*-value of < 0.001 in univariate Cox regression analysis were selected for multivariate Cox regression analysis.

### Gene set enrichment analysis

Enrichment analysis was performed using the gene set enrichment analysis (GSEA) software (version 4.1.0) and expression data extracted from TCGA–LIHC cohort to predict potential signaling pathways related to IGF2BP3 in HCC. Enrichment scores of > 0.4 and FDRs of < 0.05 were considered statistically significant. The log2 fold change values calculated using the edgeR package were used as ranking indicators^41^.

### Human samples

A total of 118 primary HCC tissues and 97 peritumoral tissues (89 pairs) were collected from patients who underwent surgical resection without radiotherapy or chemotherapy at the First Affiliated Hospital of Shihezi University from 2012 to 2019. Informed consent was obtained, and the Ethics Committee of the First Affiliated Hospital of Shihezi University approved this study. A statement to confirm that all methods were carried out in accordance with relevant guidelines and regulations.

### Immunohistochemical analysis

Sections were deparaffinized, dehydrated, and subjected to antigen retrieval. After being blocked with 5% goat serum for 1 h, the sections were incubated with primary antibodies against IGF2BP3 (ab177477, Abcam) or Ki67 (ab15580, Abcam) overnight at 4 °C. Subsequently, the sections were washed with TBST, treated with 3% H_2_O_2_, incubated with HRP-conjugated secondary antibody, and visualized using a 3,3′-diaminobenzidine kit (ZLI-9018, ZSGB­BIO). The immunoreactivity score of IGF2BP3 expression of each sample was calculated by multiplying the intensity score and extent score.

### Cell culture and transfection

Authenticated HepG2 (hepatoblastoma), Hep3B, HuH-7, and SNU-387 cells were obtained from the Cell Bank of the Chinese Academy of Sciences. MHCC97H cells were obtained from Beyotime Biotechnology. The cells were cultured in DMEM supplemented with 10% fetal bovine serum (Gibco) at 37 °C in an incubator with 5% CO_2_. A lentivirus overexpressing *IGF2BP3* and siRNAs targeting *IGF2BP3* were purchased from GenePharma (Supplementary Table 1). siRNAs were transfected using the Lipofectamine 2000 Transfection Reagent (Invitrogen) following the manufacturer’s instructions. All experiments were conducted using cells free of mycoplasma contamination.

### Quantitative reverse transcription PCR

Total RNA was extracted and synthesized to cDNA using the RevertAid First Strand cDNA Synthesis Kit (Thermo Fisher Scientific). Quantitative reverse transcription PCR (qRT-PCR) was conducted on a CFX96 Touch Real-Time PCR Detection System (Bio-Rad). The primers used for qRT-PCR are listed in Supplementary Table 2.

### CCK-8 assay

Cells were seeded in 96-well plates with clear bottoms. Cell proliferation was assessed using the Cell Counting Kit­8 (CCK­8; Dojindo) following the manufacturer’s instructions. Absorbance was quantified at a wavelength of 450 nm (OD_450_) on the Varioskan LUX Multimode Microplate Reader (Thermo Scientific).

### EdU assay

Cell proliferation was assessed via 5-ethynyl-2′-deoxyuridine (EdU) assay using the Click-iT EdU Imaging Kit (US Everbright Inc.) following the manufacturer’s instructions. Briefly, 1 × 10^5^ cells were seeded in 12-well culture plates and treated with 50 μM EdU for 2 h. After that, the cells were fixed with 4% formaldehyde for 15 min and treated with 0.5% Triton X-100 for permeabilization for 20 min. The cells were washed thrice with 3% BSA/PBS and treated with the click reaction buffer for 30 min. Subsequently, the cells were stained with Hoechst 33,342 (5 μg/mL) for 30 min and visualized under a fluorescent microscope.

### Western blotting

Equal amounts of cell lysates were separated via sodium dodecyl sulfate–polyacrylamide gel electrophoresis and transferred to polyvinylidene difluoride membranes. The membranes were cut and separately incubated with antibodies against IGF2BP3 (#ab177477, Abcam), E2F1 (SC-251, Santa Cruz Biotechnology), and β­actin (#3700, Cell Signaling Technology). Signals from the bound antibodies were detected using the Immobilon Western Chemiluminescent HRP Substrate (WBKLS0050, Millipore), and images were captured using a fluorescence/chemiluminescence imaging system (Clinx ChemiScope).

### Xenograft tumor model

BALB/c nude mice (6–8 weeks old) were acquired from SPF (Beijing) Biotechnology Co., Ltd and randomly divided into two groups. HuH-7 cells stably overexpressing IGF2BP3 or vector control (2 × 10^6^) were injected subcutaneously into each mouse until euthanized.

### Statistical analysis

Numerical data were expressed as mean ± SEM. Statistical analysis was performed using the GraphPad Prism 8 software. Data between groups were compared using the Mann–Whitney test, Wilcoxon matched­pairs signed-rank test, unpaired Student’s *t*­test, or ANOVA with post hoc tests. Survival rates were estimated using Kaplan–Meier analysis and log-rank test. The chi-square or Fisher’s exact test was used to compare categorical data. Spearman correlation coefficients were evaluated to examine the significance of the association between genes. *P < 0.05, **P < 0.01, ***P < 0.001.

### Supplementary Information

Supplementary Figures.

Supplementary Information.

## Data Availability

The data sets analyzed during this study are available from TCGA database (https://portal.gdc.cancer.gov/) and the GEO with accession numbers GSE14520, GSE90684, and GSE92220.
