# Supplementary material for: Epigenetically associated IGF2BP3 upregulation promotes cell proliferation by regulating E2F1 expression in hepatocellular carcinoma
Source: Sci Rep. 2024 Jul 11;14:16051. doi: 10.1038/s41598-024-67021-w (PMC11239653; doi:10.1038/s41598-024-67021-w)

Figure 4B: HuH-7, IGF2BP3 (left, Vector; right, IGF2BP3)


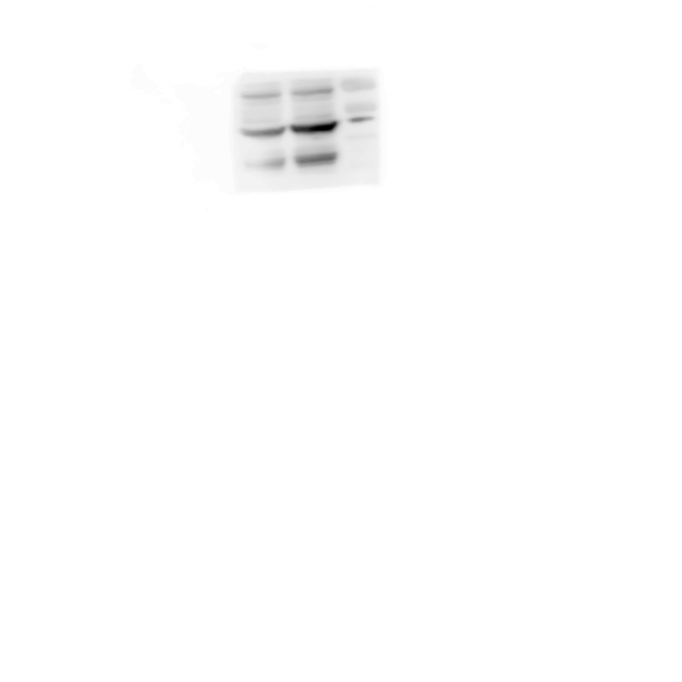


Figure 4B: HuH-7, β-actin (left, Vector; right, IGF2BP3)


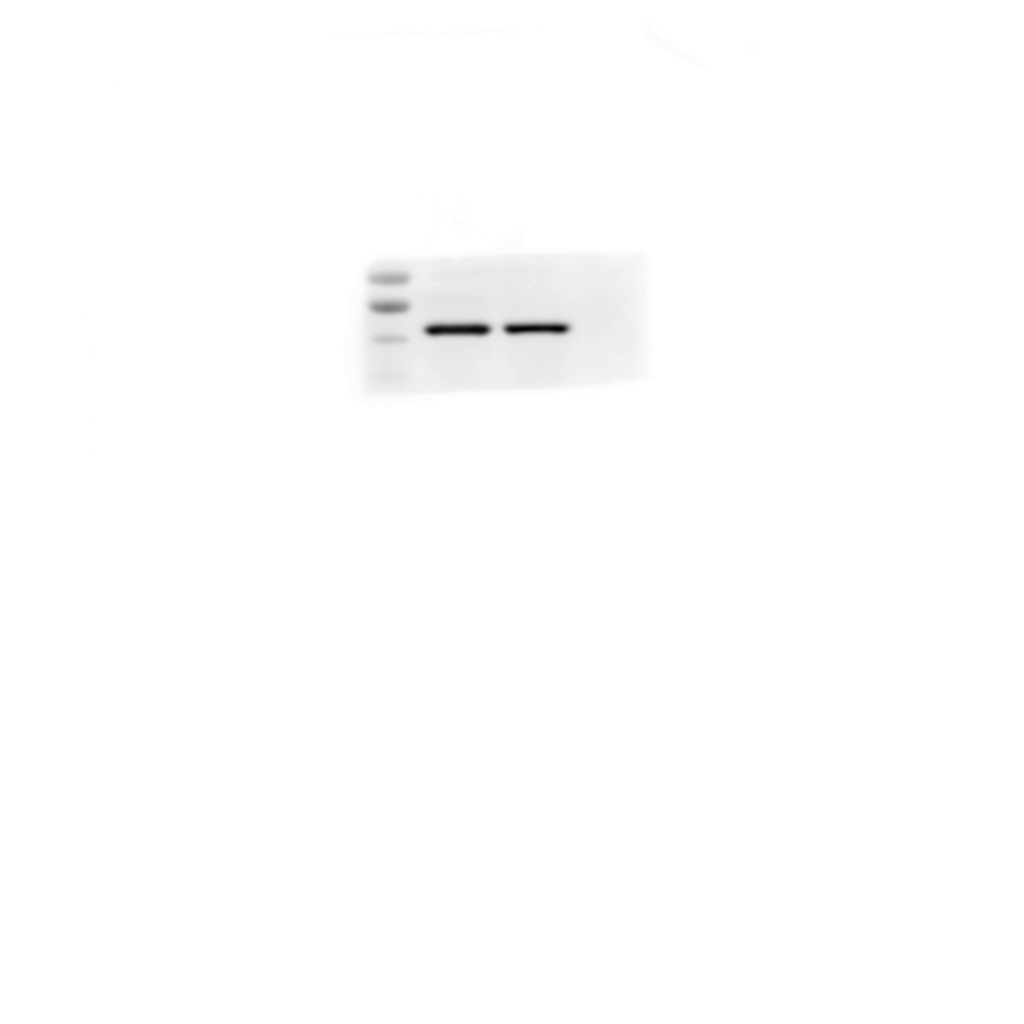


Figure 4B: MHCC97H, IGF2BP3 (left, Vector; right, IGF2BP3)


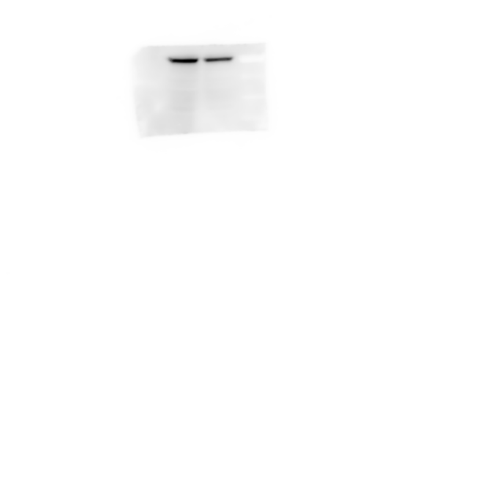


Figure 4B: MHCC97H, β-actin (left, Vector; right, IGF2BP3)


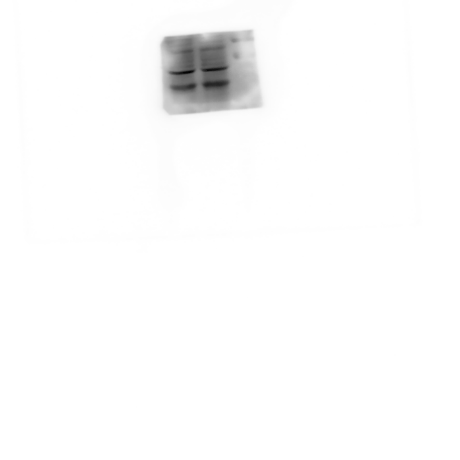


Figure 5I: Hep3B, IGF2BP3 (siCtrl, siIGF2BP3#1, siIGF2BP3#2, siIGF2BP3#3)


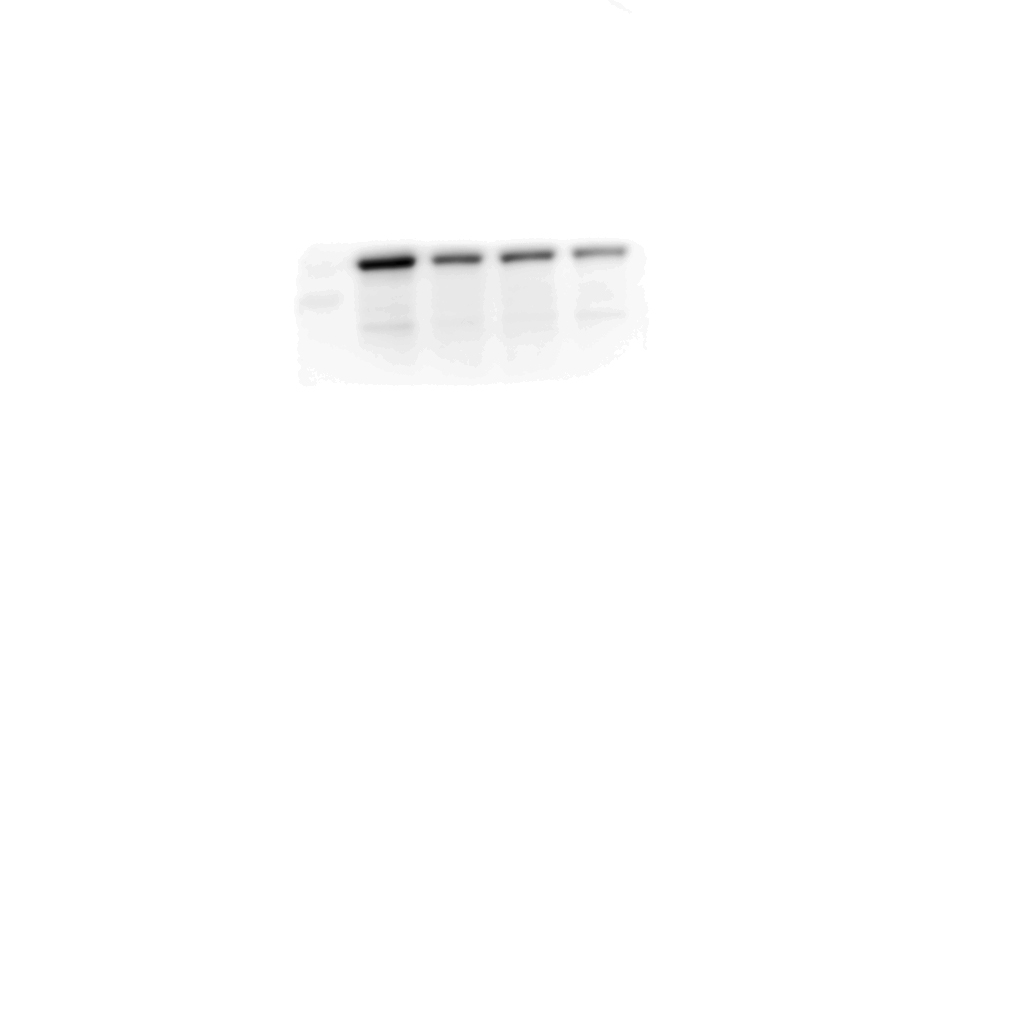


Figure 5I: Hep3B, E2F1 (siCtrl, siIGF2BP3#1, siIGF2BP3#2, siIGF2BP3#3)


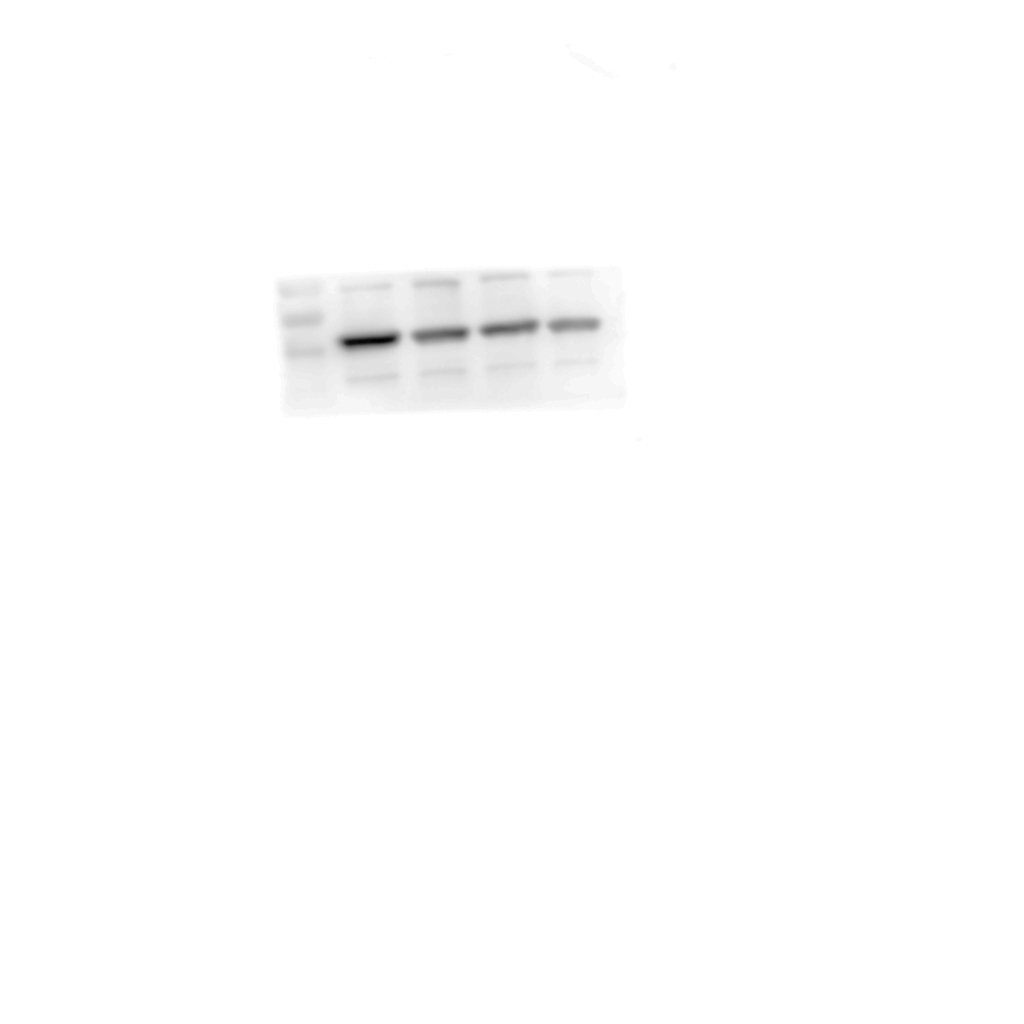


Figure 5I: Hep3B, β-actin (siCtrl, siIGF2BP3#1, siIGF2BP3#2, siIGF2BP3#3)


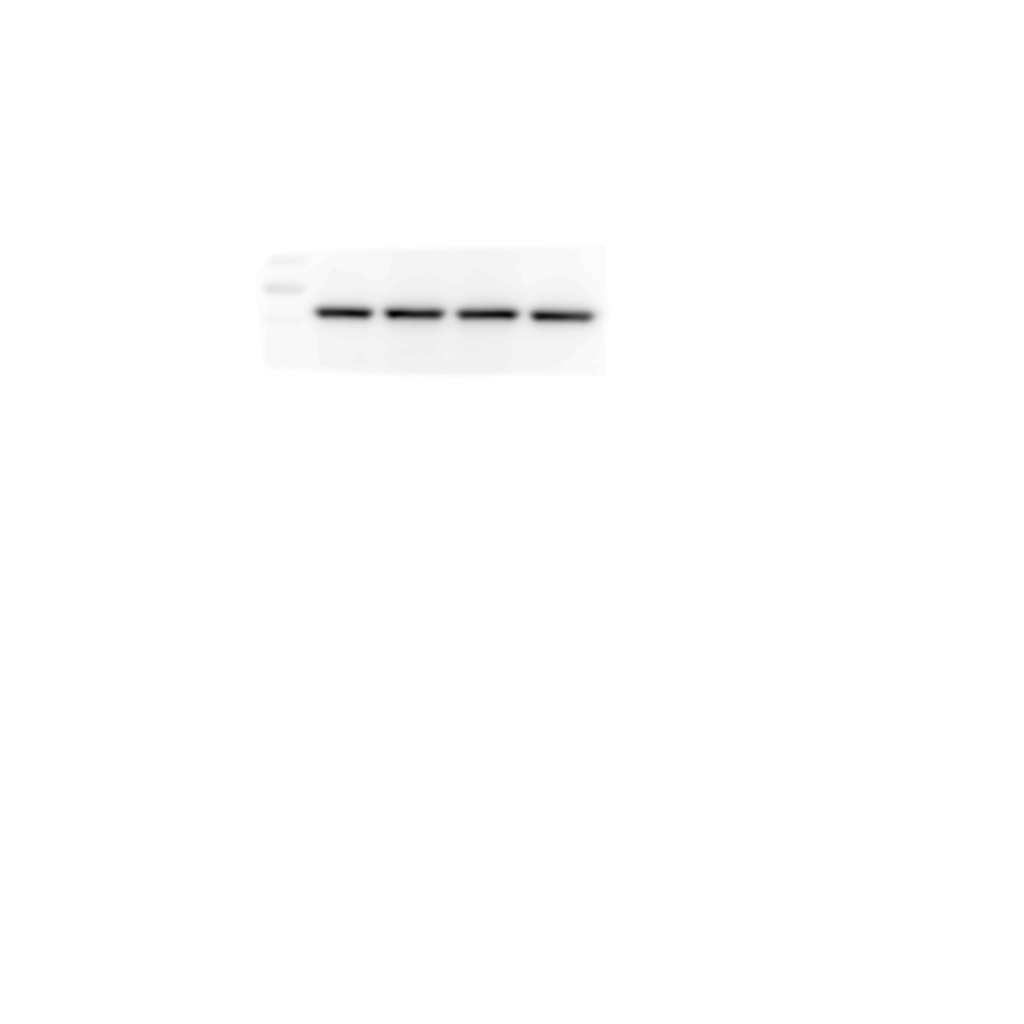


Figure 5I: HepG2, IGF2BP3 (siCtrl, siIGF2BP3#1, siIGF2BP3#2, siIGF2BP3#3)


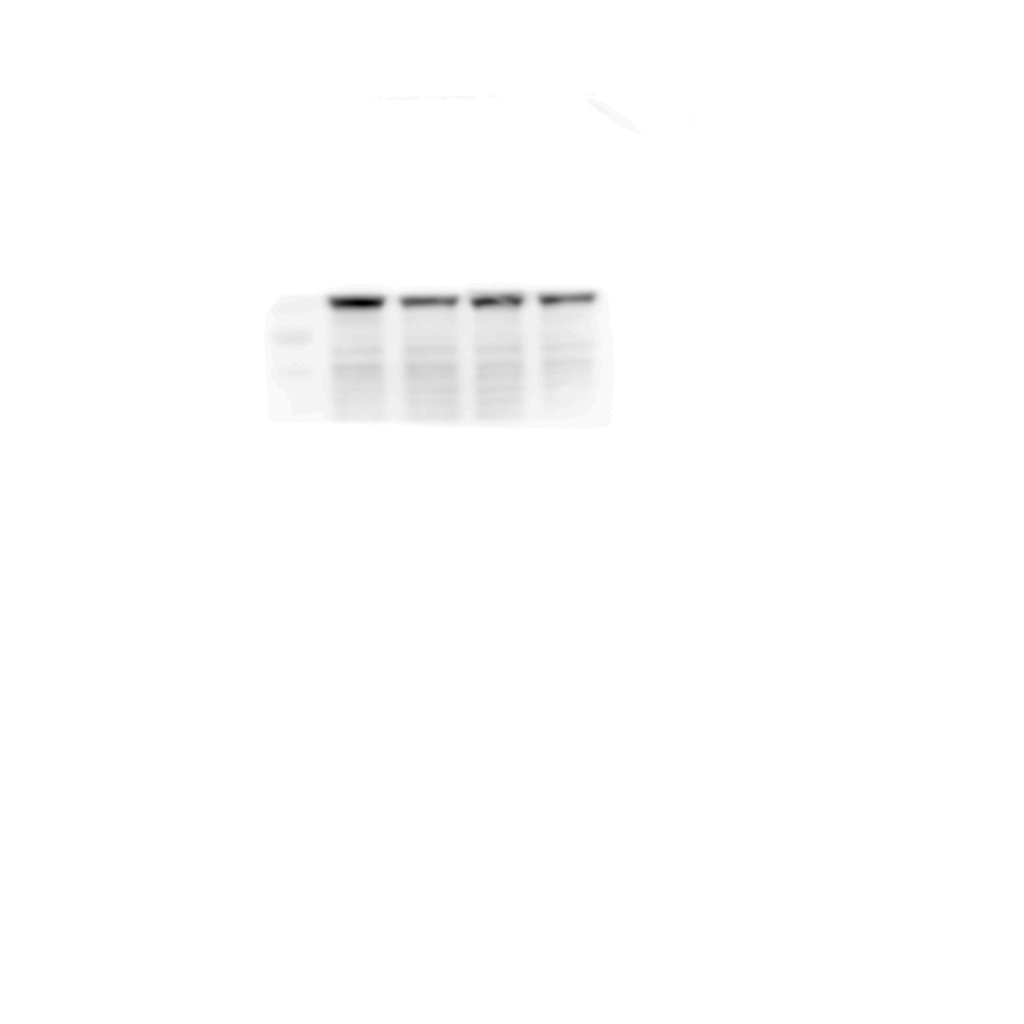


Figure 5I: HepG2, E2F1 (siCtrl, siIGF2BP3#1, siIGF2BP3#2, siIGF2BP3#3)


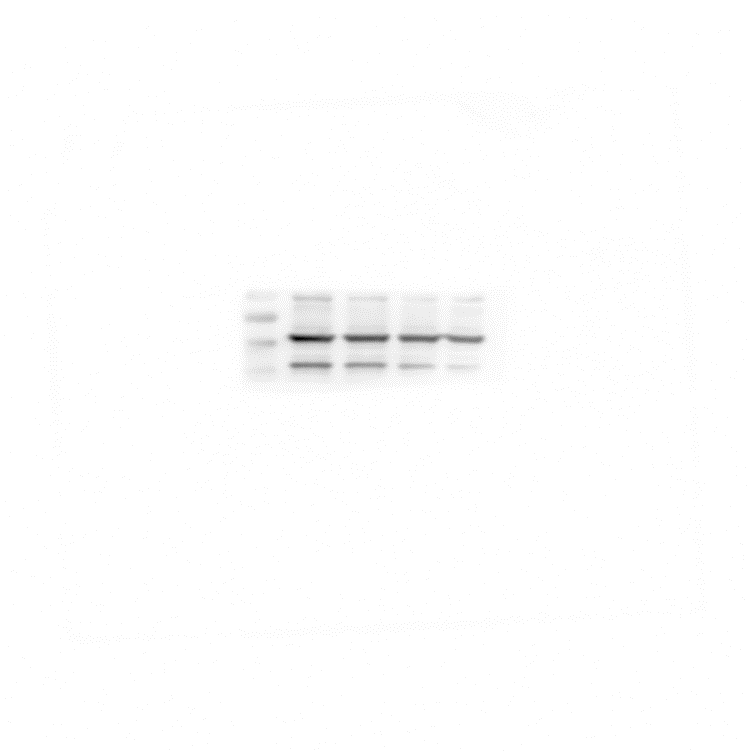


Figure 5I: HepG2, β-actin (siCtrl, siIGF2BP3#1, siIGF2BP3#2, siIGF2BP3#3)


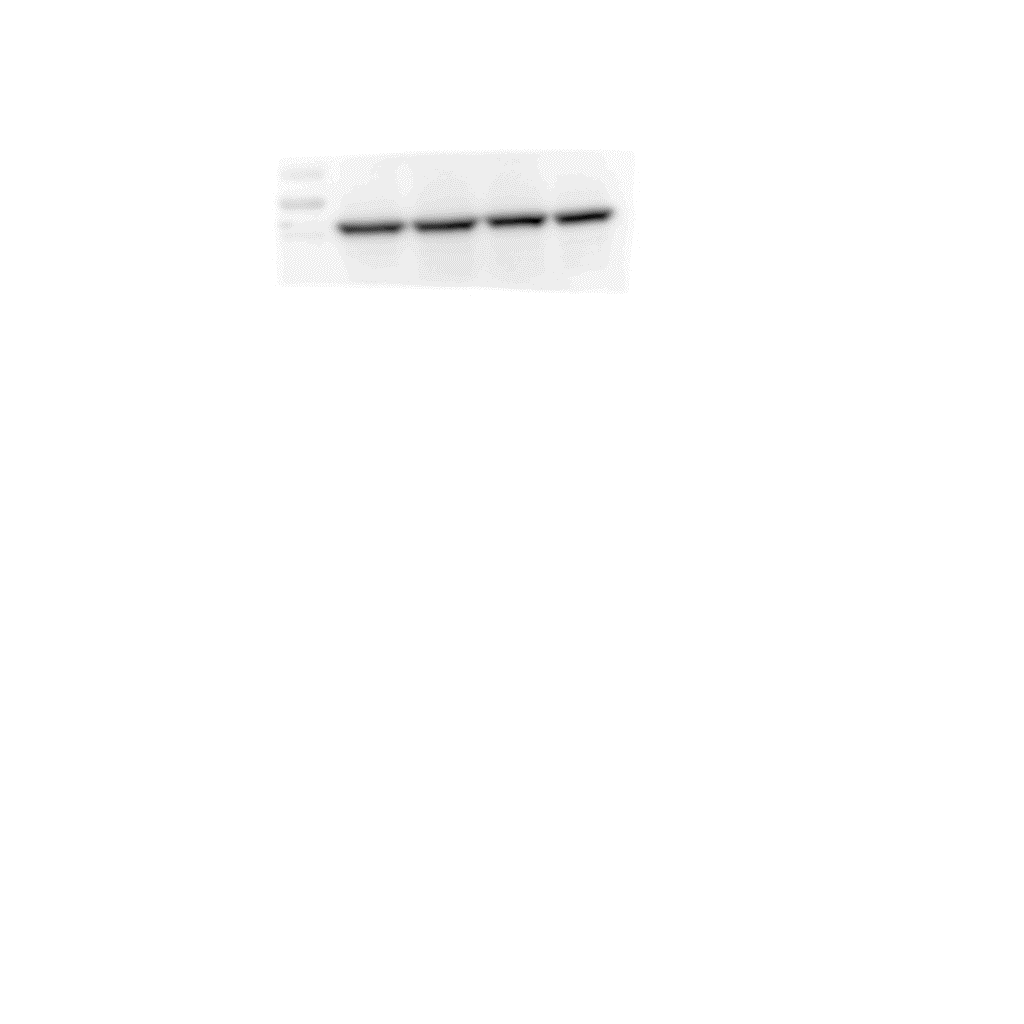


Figure 5K: HuH-7, E2F1 (Vector + siCtrl, IGF2BP3 + siCtrl, IGF2BP3 + siE2F1#1, IGF2BP3 + siE2F2#2)


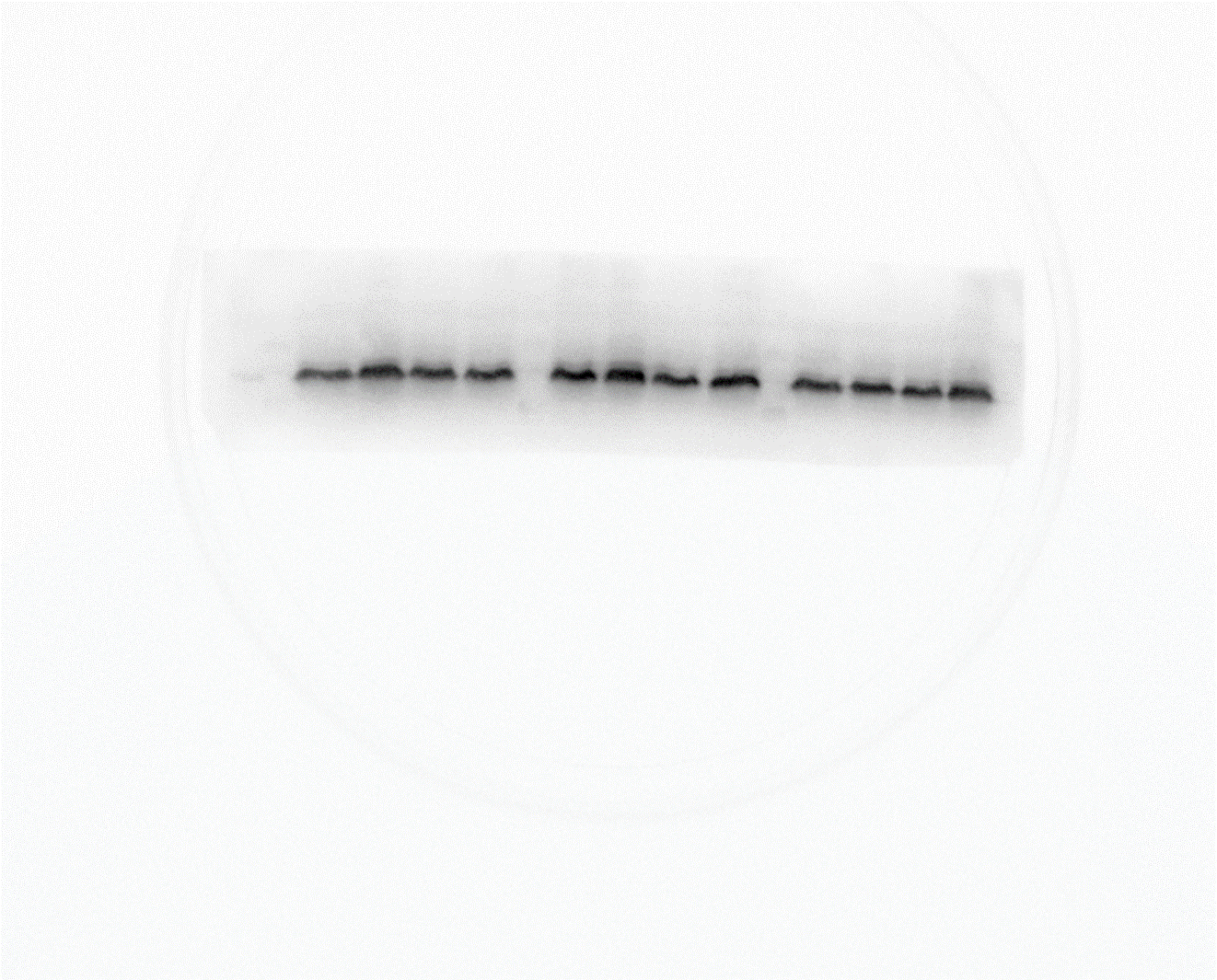


Figure 5K: HuH-7, GAPDH (Vector + siCtrl, IGF2BP3 + siCtrl, IGF2BP3 + siE2F1#1, IGF2BP3 + siE2F2#2)


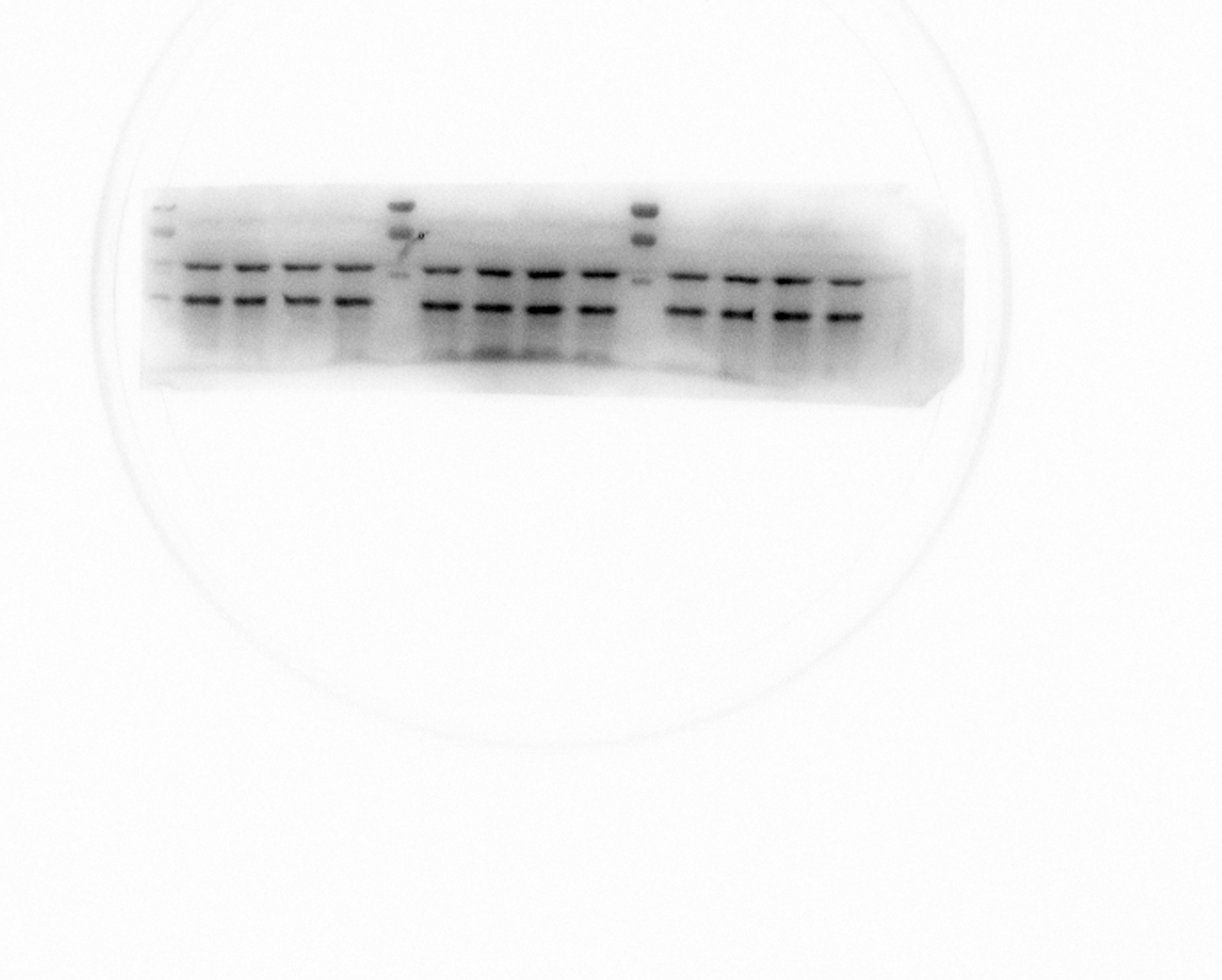


Figure 6C: MHCC97H, IGF2BP3 (Vehicle, Decitabine)


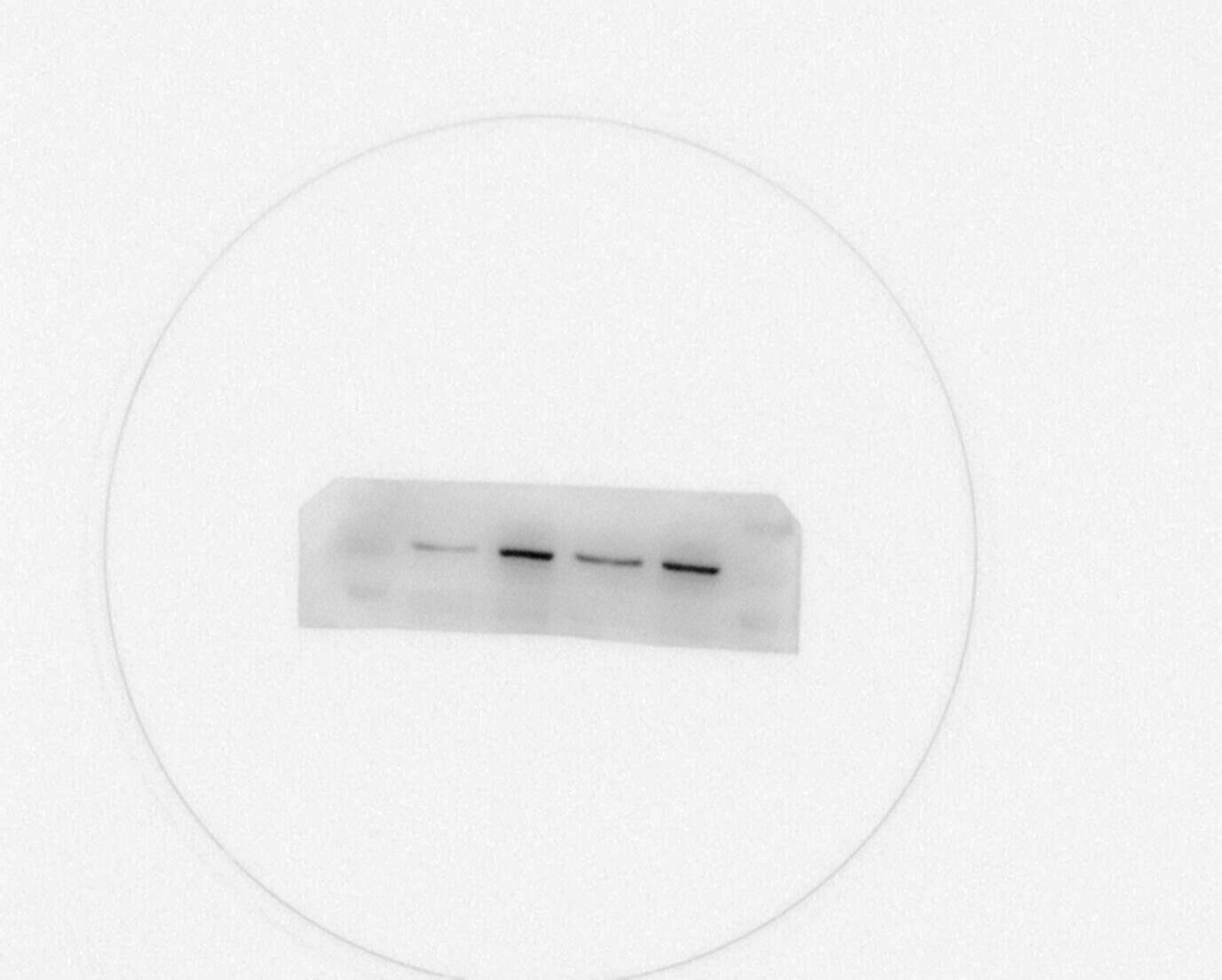


Figure 6C: MHCC97H, GAPDH (Vehicle, Decitabine)


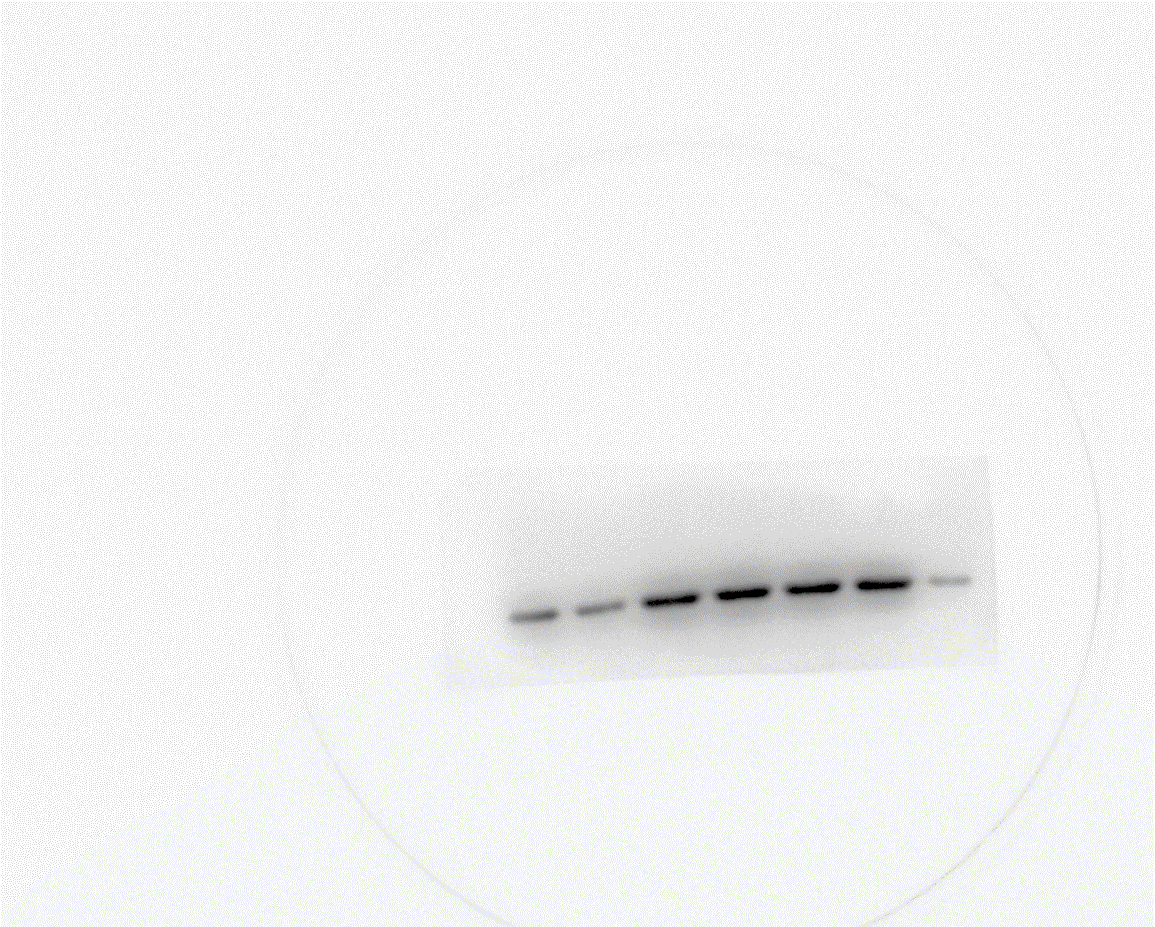

Supplement: Supplementary file 1 — Supplementary Figures. [file 41598_2024_67021_MOESM1_ESM.docx]
