# Supplementary material for: Epigenetically associated IGF2BP3 upregulation promotes cell proliferation by regulating E2F1 expression in hepatocellular carcinoma
Source: Sci Rep. 2024 Jul 11;14:16051. doi: 10.1038/s41598-024-67021-w (PMC11239653; doi:10.1038/s41598-024-67021-w)
Supplement: Supplementary file 2 — Supplementary Information. [file 41598_2024_67021_MOESM2_ESM.docx]

**
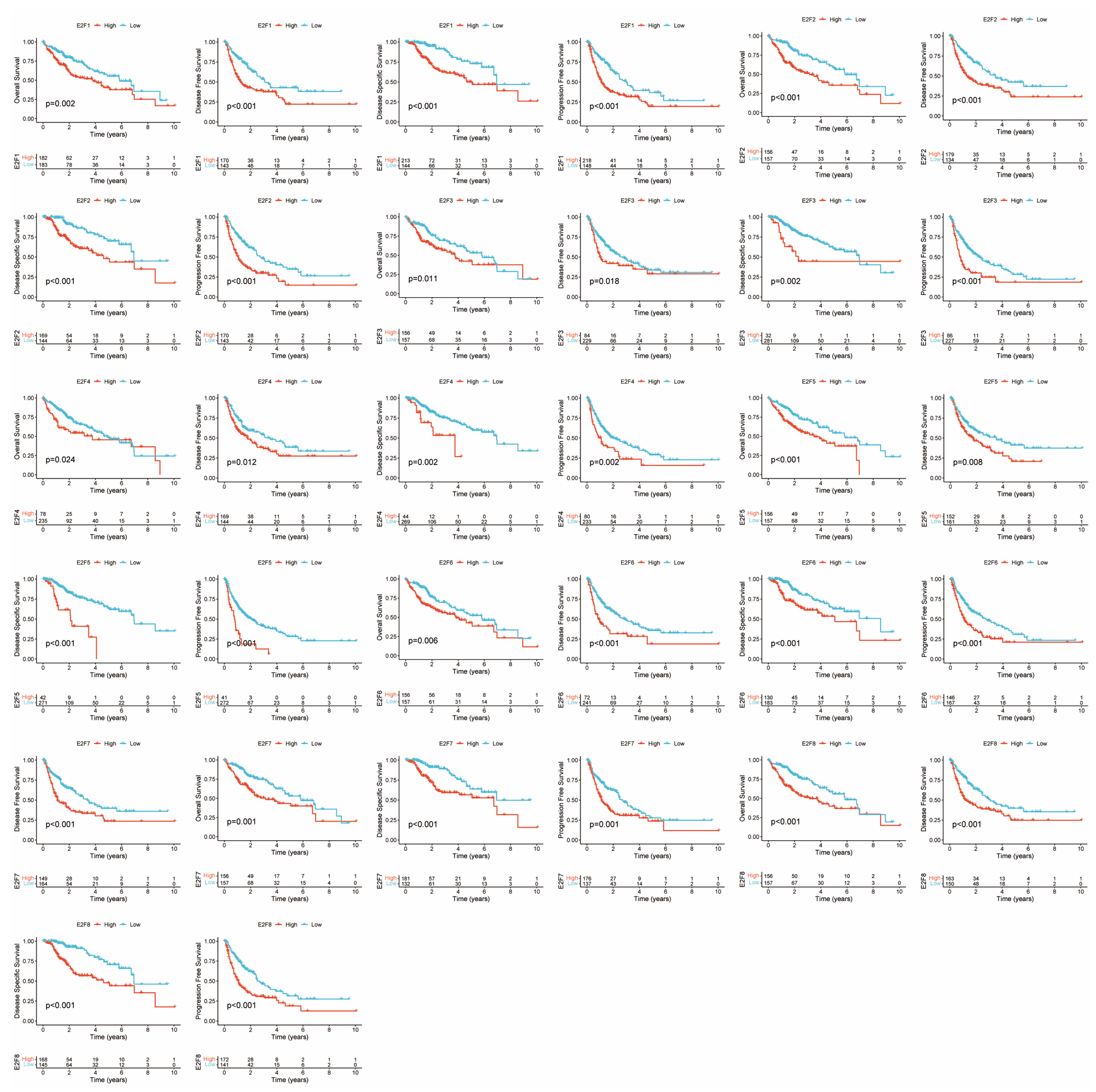
**

**Supplement Figure 1.** Kaplan-­Meier analyses of E2F expression levels and survival of patients from TCGA-­LIHC.

**
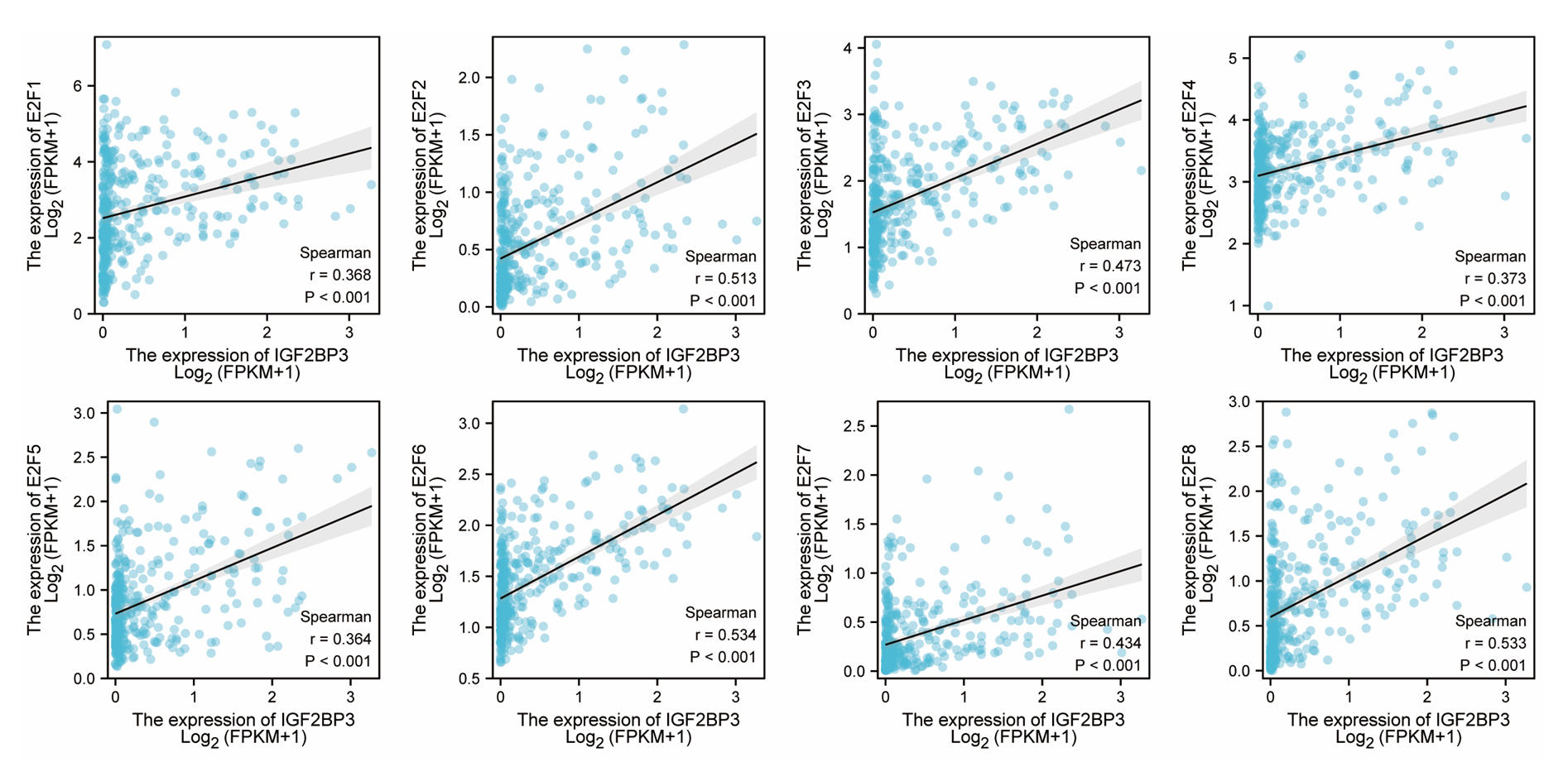
**

**Supplement Figure 2.** Correlation between the expression of IGF2BP3 and E2Fs in TCGA-LIHC cohort.

**Supplementary Table 1. Sequences for gene knockdown.**

| **Symbol** | **sense (5'-3')** | **antisense (5'-3')** |
| --- | --- | --- |
| siCtrl | UUCUCCGAACGUGUCACGUTT | ACGUGACACGUUCGGAGAATT |
| siIGF2BP3#1 | GGAUUCGGAAACUUCAGAUTT | AUCUGAAGUUUCCGAAUCCTT |
| siIGF2BP3#2 | CACCUUGAAAGUAGCCUAUTT | AUAGGCUACUUUCAAGGUGTT |
| siIGF2BP3#3 | GCUGCUGAGAAGUCGAUUATT | UAAUCGACUUCUCAGCAGCTT |
| siMETTL3#1 | GGUUGGUGUCAAAGGAAAUTT | AUUUCCUUUGACACCAACCTT |
| siMETTL3#2 | GGUGACUGCUCUUUCCUUATT | UAAGGAAAGAGCAGUCACCTT |
| siMETTL3#3 | GCAAGAAUUCUGUGACUAUTT | AUAGUCACAGAAUUCUUGCTT |
| siMETTL14#1 | GGAUGAAGGAGAGACAGAUTT | AUCUGUCUCUCCUUCAUCCTT |
| siMETTL14#2 | GCAGCACCUCGAUCAUUUATT | UAAAUGAUCGAGGUGCUGCTT |
| siMETTL14#3 | CCUGGGAAGACUAAGACUUTT | AAGUCUUAGUCUUCCCAGGTT |

**Supplementary Table 2. Primers for qRT**-**PCR.**

| **Gene** | **Primer** |
| --- | --- |
| β-actin | For: 5'-AACCGCGAGAAGATGACCCAG-3' |
|  | Rev: 5'-GGATAGCACAGCCTGGATAGCAA-3' |
| IGF2BP3 | For: 5'-ATCGAGGCGCTTTCAGGTAA-3' |
|  | Rev: 5'-TCCCACTGTAAATGAGGCGG-3' |
| E2F1 | For: 5'-GCCATCCAGGAAAAGGTGTGA-3' |
|  | Rev: 5'-TTCAGGTCGACGACACCGT-3' |
| E2F2 | For: 5'-CAACATCCAGTGGGTAGGCA-3' |
|  | Rev: 5'-TGCTCCGTGTTCATCAGCTC-3' |
| E2F3 | For: 5'-ACAACGTCCAATGGATGGGC-3' |
|  | Rev: 5'-CAGGGTGCAGCTTTGGATCA-3' |
| E2F4 | For: 5'-TCCGGACCCAACCCTTCTAC-3' |
|  | Rev: 5'-GAGCTCATGCACTCTCGTGT-3' |
| E2F5 | For: 5'-TGATACTTTGGCTGTGAGGCA-3' |
|  | Rev: 5'-CAGCACCTACACCTTTCCACTG-3' |
| E2F6 | For: 5'-GTTCCAGCTCCCAGAGAAGACT-3' |
|  | Rev: 5'-TACTGGTCTGACCCTGCTCC-3' |
| E2F7 | For: 5'-GATCGATCAAGGATGGCCCC-3' |
|  | Rev: 5'-TTCCGCTTGCTGTCTGTCAA-3' |
| E2F8 | For: 5'-TCCCAACCAAGTCGAAAAGAGA-3' |
|  | Rev: 5'-TGCGTCGACGTTCAACATTAAG-3' |
| METTL3 | For: 5'-ACACTGCTTGGTTGGTGTCA-3' |
|  | Rev: 5'-CGAACCTCAGCTACGATCACA-3' |
| METTL14 | For: 5'-CAAAGGAACACTGCCTCATGG-3' |
|  | Rev: 5'-CCAGCCTGGTCGAATTGTACT-3' |

**Supplementary Table 3. GO enrichment analysis of aberrantly expressed RBPs.**

|  | **GO term** | ***P* value** | **FDR** |
| --- | --- | --- | --- |
| **Down-regulated** |  |  |  |
| BP | regulation of translation | 2.32E-14 | 1.15E-11 |
|  | regulation of mRNA metabolic process | 4.37E-10 | 1.09E-07 |
|  | negative regulation of translation | 5.40E-09 | 8.95E-07 |
|  | negative regulation of cellular amide metabolic process | 1.00E-08 | 1.06E-06 |
|  | regulation of mRNA processing | 1.07E-08 | 1.06E-06 |
|  | cytoplasmic translation | 3.26E-08 | 2.70E-06 |
| CC | messenger ribonucleoprotein complex | 8.04E-07 | 3.94E-05 |
|  | cytoplasmic ribonucleoprotein granule | 5.38E-06 | 0.000112 |
|  | ribonucleoprotein granule | 6.84E-06 | 0.000112 |
|  | cytoplasmic stress granule | 1.26E-05 | 0.000155 |
|  | mRNA cap binding complex | 0.00024 | 0.002348 |
|  | RNA cap binding complex | 0.00033 | 0.002692 |
| MF | single-stranded RNA binding | 1.58E-13 | 1.39E-11 |
|  | translation regulator activity | 8.54E-12 | 3.76E-10 |
|  | mRNA 3'-UTR binding | 8.93E-11 | 2.62E-09 |
|  | translation repressor activity | 1.85E-09 | 2.93E-08 |
|  | mRNA 3'-UTR AU-rich region binding | 1.85E-09 | 2.93E-08 |
|  | AU-rich element binding | 2.26E-09 | 2.93E-08 |
| **Up-regulated** |  |  |  |
| BP | RNA splicing | 4.06E-36 | 3.93E-33 |
|  | mRNA splicing, via spliceosome | 4.18E-25 | 1.23E-22 |
|  | RNA splicing, via transesterification reactions | 5.06E-25 | 1.23E-22 |
|  | regulation of mRNA metabolic process | 3.53E-19 | 6.84E-17 |
|  | mRNA transport | 5.95E-18 | 9.61E-16 |
|  | nucleic acid transport | 3.83E-16 | 4.64E-14 |
| CC | cytoplasmic stress granule | 1.11E-08 | 1.11E-06 |
|  | spliceosomal complex | 3.96E-08 | 1.98E-06 |
|  | cytoplasmic ribonucleoprotein granule | 2.07E-07 | 6.91E-06 |
|  | ribonucleoprotein granule | 2.96E-07 | 7.39E-06 |
|  | mRNA cleavage and polyadenylation specificity factor complex | 5.25E-07 | 1.05E-05 |
| MF | mRNA cleavage factor complex | 1.24E-06 | 2.07E-05 |
|  | mRNA 3'-UTR binding | 1.10E-15 | 1.41E-13 |
|  | poly(U) RNA binding | 5.72E-08 | 3.30E-06 |
|  | translation regulator activity | 8.75E-08 | 3.30E-06 |
|  | poly-pyrimidine tract binding | 1.02E-07 | 3.30E-06 |
|  | single-stranded RNA binding | 1.67E-06 | 4.30E-05 |
|  | poly(A) binding | 2.03E-06 | 4.37E-05 |

**Supplementary Table 4. KEGG pathway enrichment analysis of aberrantly expressed RBPs.**

| **KEGG pathway** | ***P* value** | **FDR** |
| --- | --- | --- |
| **Down-regulated** |  |  |
| mRNA surveillance pathway | 3.27E-05 | 0.000686 |
| RNA transport | 0.000393 | 0.004128 |
| Progesterone-mediated oocyte maturation | 0.000907 | 0.006351 |
| Oocyte meiosis | 0.001894 | 0.009941 |
| Ribosome biogenesis in eukaryotes | 0.019335 | 0.081208 |
| Spliceosome | 0.033176 | 0.110627 |
| 2-Oxocarboxylic acid metabolism | 0.036876 | 0.110627 |
| Influenza A | 0.044185 | 0.115986 |
| **Up-regulated** |  |  |
| mRNA surveillance pathway | 4.09E-17 | 8.18E-16 |
| RNA transport | 1.22E-10 | 1.22E-09 |
| RNA degradation | 4.33E-06 | 2.89E-05 |
| Spliceosome | 8.85E-05 | 0.000443 |
| Non-homologous end-joining | 0.040924 | 0.163697 |
